# Potential for redistribution of post‐moult habitat for *Eudyptes* penguins in the Southern Ocean under future climate conditions

**DOI:** 10.1111/gcb.16500

**Published:** 2022-11-09

**Authors:** Cara‐Paige Green, David B. Green, Norman Ratcliffe, David Thompson, Mary‐Anne Lea, Alastair M. M. Baylis, Alexander L. Bond, Charles‐André Bost, Sarah Crofts, Richard J. Cuthbert, Jacob González‐Solís, Kyle W. Morrison, Maud Poisbleau, Klemens Pütz, Andrea Raya Rey, Peter G. Ryan, Paul M. Sagar, Antje Steinfurth, Jean‐Baptiste Thiebot, Megan Tierney, Thomas Otto Whitehead, Simon Wotherspoon, Mark A. Hindell

**Affiliations:** ^1^ Institute for Marine and Antarctic Studies University of Tasmania Hobart Tasmania Australia; ^2^ ARC Australian Centre for Excellence in Antarctic Science Institute for Marine and Antarctic Studies, University of Tasmania Hobart Tasmania Australia; ^3^ British Antarctic Survey Cambridge UK; ^4^ National Institute of Water and Atmospheric Research Ltd. Hataitai Wellington New Zealand; ^5^ South Atlantic Environmental Research Institute Stanley Falkland Islands; ^6^ Macquarie University Sydney New South Wales Australia; ^7^ RSPB Centre for Conservation Science Royal Society for the Protection of Birds The Lodge Sandy UK; ^8^ Bird Group Natural History Museum Ting UK; ^9^ Centre d'Etudes Biologiques de Chizé UMR7372 CNRS‐La Rochelle Université Villiers en Bois France; ^10^ Falklands Conservation Stanley Falkland Islands; ^11^ Royal Society for the Protection of Birds Centre for Conservation Science Cambridge UK; ^12^ World Land Trust Blyth House Halesworth UK; ^13^ Institut de Recerca de la Biodiversitat (IRBio) and Departament de Biologia Evolutiva Ecologia i Ciències Ambientals Universitat de Barcelona Barcelona Spain; ^14^ Behavioural Ecology and Ecophysiology Group Department of Biology University of Antwerp Wilrijk Belgium; ^15^ Antarctic Research Trust Bremervörde Germany; ^16^ CADIC‐CONICET WCS Representación Argentina Ushuaia Argentina; ^17^ FitzPatrick Institute of African Ornithology DST‐NRF Centre of Excellence University of Cape Town Rondebosch South Africa; ^18^ National Institute of Water and Atmospheric Research Ltd. Christchurch New Zealand; ^19^ Graduate School of Fisheries Sciences Hokkaido University Hakodate Japan; ^20^ Joint Nature Conservation Committee Peterborough UK; ^21^ Australian Antarctic Division Department of Agriculture, Water and the Environment Australian Antarctic Division Kingston Tasmania Australia

**Keywords:** climate change, habitat preference models, migration, overwinter, species redistributions, Subantarctic penguins

## Abstract

Anthropogenic climate change is resulting in spatial redistributions of many species. We assessed the potential effects of climate change on an abundant and widely distributed group of diving birds, *Eudyptes* penguins, which are the main avian consumers in the Southern Ocean in terms of biomass consumption. Despite their abundance, several of these species have undergone population declines over the past century, potentially due to changing oceanography and prey availability over the important winter months. We used light‐based geolocation tracking data for 485 individuals deployed between 2006 and 2020 across 10 of the major breeding locations for five taxa of *Eudyptes* penguins. We used boosted regression tree modelling to quantify post‐moult habitat preference for southern rockhopper (*E. chrysocome*), eastern rockhopper (*E. filholi*), northern rockhopper (*E. moseleyi*) and macaroni/royal (*E. chrysolophus* and *E. schlegeli*) penguins. We then modelled their redistribution under two climate change scenarios, representative concentration pathways RCP4.5 and RCP8.5 (for the end of the century, 2071–2100). As climate forcings differ regionally, we quantified redistribution in the Atlantic, Central Indian, East Indian, West Pacific and East Pacific regions. We found sea surface temperature and sea surface height to be the most important predictors of current habitat for these penguins; physical features that are changing rapidly in the Southern Ocean. Our results indicated that the less severe RCP4.5 would lead to less habitat loss than the more severe RCP8.5. The five taxa of penguin may experience a general poleward redistribution of their preferred habitat, but with contrasting effects in the (i) change in total area of preferred habitat under climate change (ii) according to geographic region and (iii) the species (macaroni/royal vs. rockhopper populations). Our results provide further understanding on the regional impacts and vulnerability of species to climate change.

## Graphical abstract

Ongoing changes to the marine environment due to anthropogenic climate change have the potential to cause large scale spatial redistributions to nonbreeding habitat of five taxa of Eudyptes penguin. These penguins may experience a poleward shift in habitat which could have consequences for time spent travelling and searching for sufficient food. Our findings add to understanding of regional impacts and vulnerability of species to climate change.

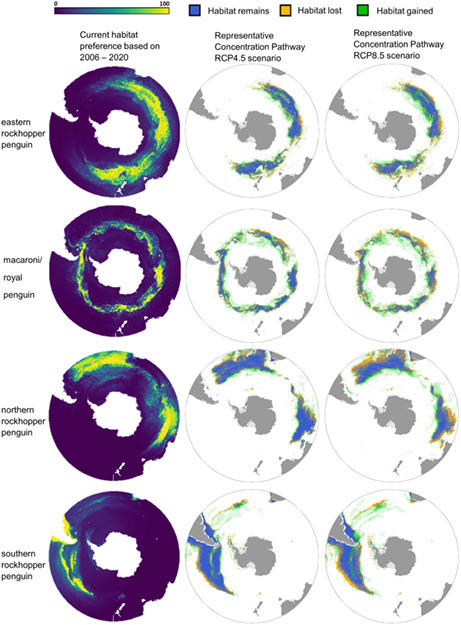

## 1 INTRODUCTION

Anthropogenic climate change is resulting in redistributions of species worldwide with marine species redistributing poleward at a rate six times faster than terrestrial species (Lenoir et al., 2020). The Southern Ocean (defined here as including the southern zones of the Indian, Atlantic and Pacific Oceans, from the Subtropical Front to the Antarctic continent; Deacon, 1982) is one of the most rapidly warming oceans on the planet (Sallée, 2018). Here, abundant populations of marine predators play a key structuring role in marine ecosystems (Bestley et al., 2020), and act as bio‐indicators of the ecosystem state (Hazen et al., 2019). Many marine predators may be threatened by the rapid changes taking place in the Southern Ocean (Barbraud et al., 2011; Constable et al., 2014; Le Bohec et al., 2008; Trathan et al., 2007). Despite this, the first Marine Ecosystem Assessment for the Southern Ocean (MEASO) highlighted the lack of knowledge on how marine predators will be redistributed across their ranges (Bestley et al., 2020; but see Cristofari et al., 2018; Hindell et al., 2020; Reisinger et al., 2022). The MEASO project aims to collate research and assess trends on Southern Ocean ecosystems under climate change (with the aim of enabling policy makers to achieve consensus in adapting their management strategies to ecosystem change). Conservation and management of these animals relies on understanding their distributions, how these relate to the bio‐physical environment (Reisinger et al., 2018); and how these distributions may alter in a changing marine environment (Pecl et al., 2017). Predicting how distributions of species may change is critical in predicting their potential for adapting to climate‐related environmental change (Jenouvrier et al., 2014; Rose et al., 2010).

Marine predators, such as penguins, are limited in their capacity to respond to rapid changes in their environment and the resulting mismatch in the distribution of foraging habitat and prey (Bost et al., 2015; Dias et al., 2019; Grémillet & Boulinier, 2009; Morgenthaler et al., 2018). Penguins represent nearly 90% of the avian biomass in the Southern Ocean (Croxall & Lishman, 1987). However, populations of several species are declining and are predicted to decline further as warming of the ocean continues (Barbraud et al., 2008; Boersma et al., 2020; Jenouvrier et al., 2020; Ropert‐Coudert et al., 2019). Penguin species are particularly vulnerable to changes in preferred habitat or prey redistribution compared to flying birds because they cannot as easily cover large distances to forage. Indeed, metabolic rates of swimming penguins are equivalent to 2.5 times those of soaring albatrosses of a similar body mass (Alexander, 2002). Migrations are energetically costly, and penguins can only perform these post‐moult migration round trips of ~10,000 km through high energetic payoffs over this period (Alexander, 2002). Additionally, penguins are restricted by the limited number of breeding/moulting sites accessible to them which reduce their capacity to relocate colonies if resources move (Cristofari et al., 2018).

The penguin genus *Eudyptes* (crested penguins) consists of eight taxa (seven IUCN recognized species) that breed in locations from the subtropics to the Antarctica peninsula. This penguin assemblage is the main avian consumer in the Southern Ocean in terms of biomass consumption, eating millions of tonnes of prey (including euphausiids, myctophids and cephalopods) annually (Brooke, 2004). Yet, despite their widespread abundance, several species have undergone population declines over the past century (Allinson, 2018). *Eudyptes* penguins are highly philopatric (Thiebot, Authier, et al., 2014; Thiebot, Cherel, et al., 2014; Williams & Rodwell, 1992). They perform two migrations annually: the pre‐moult migration occurs immediately after breeding and lasts 1–2 months before their catastrophic moult (when they moult all their feathers at once), and the post‐moult migration when they spend 4–6 months overwintering at sea, covering vast distances. During the post‐moult migration, the first and last months are particularly important as individuals undergo hyperphagia (periods of intensive foraging) during which they recover condition following their catastrophic moult (throughout which they fast for 4–5 weeks) and gain substantial fat and muscle reserves in preparation for breeding (Green et al., 2005; Ratcliffe, Crofts, et al., 2014; Thiebot, Cherel, et al., 2011).

Direct threats to *Eudyptes* penguin include oiling and bycatch (Crawford et al., 2017; Guggenheim & Glass, 2014; Pütz et al., 2002). However, population declines of marine predators are also mediated by the changing biotic and abiotic oceanographic conditions that influence the distribution and abundance of marine resources (Péron et al., 2012; Trathan et al., 2007). Population changes of *Eudyptes* penguins, in particular, have been caused by changes in oceanography and prey availability which can impact on nonbreeding survival and on breeding success (Crawford et al., 2008; Crawford & Dyer, 2006; Horswill et al., 2016; Morgenthaler et al., 2018). Therefore, it is important to predict how future conditions under climate change might affect the availability and extent of preferred nonbreeding habitats (which reflects where preferred prey will be distributed) and hence post‐moult distributions of *Eudyptes* penguins.

Here, we investigated the habitat preference and potential redistribution of preferred habitat in response to climate change for five *Eudyptes* taxa, during their post‐moult period. We quantified current physical habitat characteristics of the southern rockhopper (*E. chrysocome*), eastern rockhopper (*E. chrysocome filholi*), northern rockhopper (*E. moseleyi*) and macaroni/royal (*E. chrysolophus* and *E. schlegeli*) penguins in the Southern Ocean. In this study, we define the Southern Ocean to include waters ranging from 30 °S to Antarctica following Deacon (1982) and Talley (2011) encompassing the breeding islands and foraging ranges of northern rockhoppers. Thereafter, we identified potential future habitat analogues under two climate change scenarios, Representative Concentration Pathways RCP4.5 (our current projection) and RCP8.5 (worst case scenario). RCP4.5 and RCP8.5 correspond to medium and high radiative forcing, respectively. Radiative forcing is the measure of the amount of downward‐directed radiant energy upon the Earth's surface from greenhouse gas emissions, aerosol emissions and solar irradiance (Portner, 2019). RCP4.5 projects global temperatures increasing by 1.1–2.6°C and a mean sea level rise of 0.47 m, by the year 2100 and RCP8.5 projects global temperatures increasing by 3.0–12.6°C and a mean sea level rise of 0.62 m, by the year 2100 (Meinshausen et al., 2011; Pielke Jr. et al., 2022). Our aims were to (1) use multi‐species and multi‐population post‐moult tracking data to infer post‐moult habitat preference for five taxa of *Eudyptes* penguins from nearly all major breeding sites across the Southern Ocean; (2) use two greenhouse gas concentration pathways, RCP4.5 and RCP8.5, to model future potential redistribution of habitat (at the end of the century, 2071–2100); and (3) quantify the magnitude of habitat change per MEASO‐defined region (Grant et al., 2021; McCormack et al., 2021) for both RCP scenarios for each taxon.

## 2 METHODS

### 2.1 Breeding distributions of study species

**FIGURE 1:**
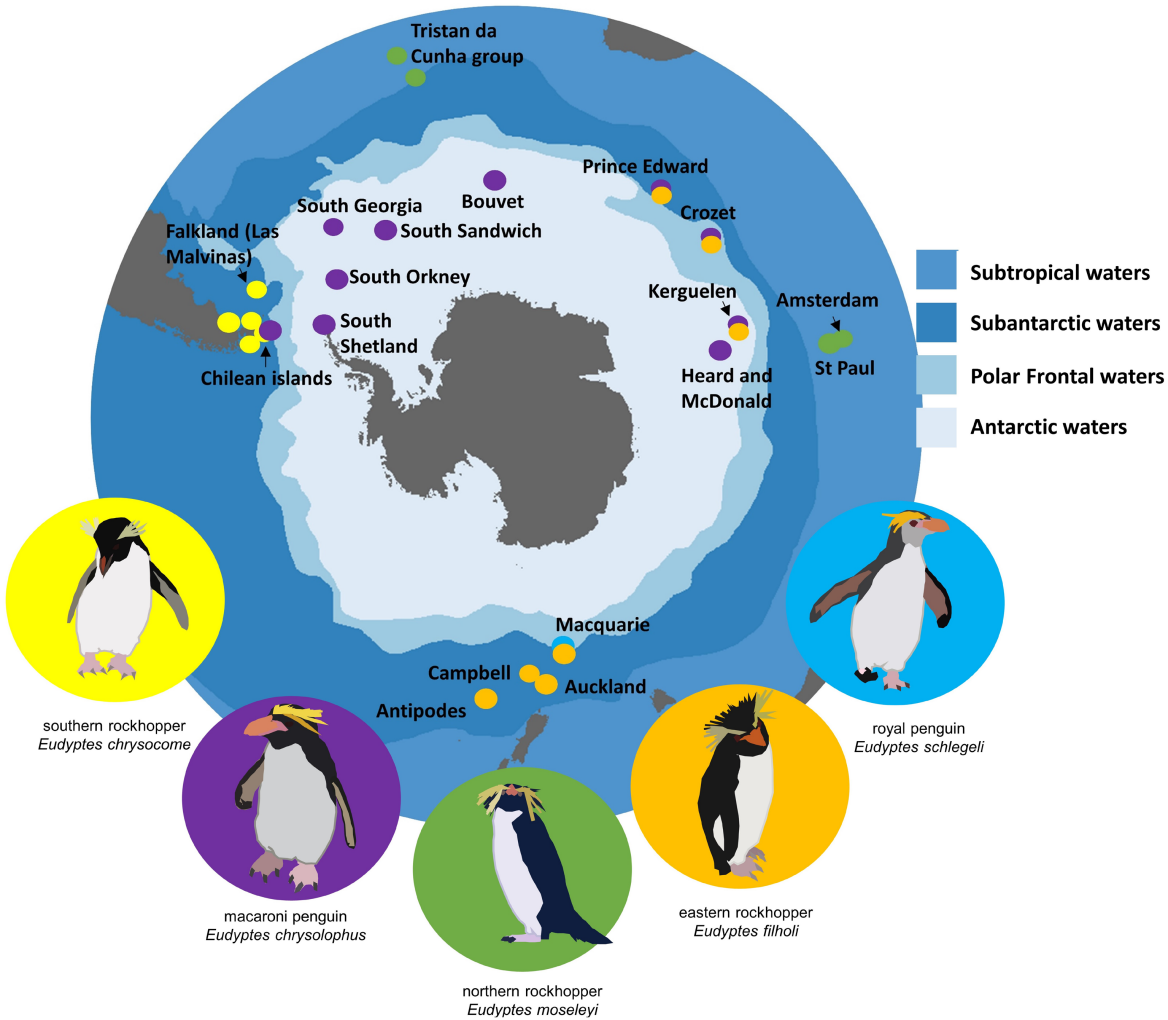
Distribution of breeding sites of the five focal *Eudyptes* penguin taxa across the Southern Ocean: eastern rockhopper penguins, macaroni/royal penguins, northern rockhopper penguins and southern rockhopper penguins.

Currently, there are two recognized species of rockhopper penguins (IUCN, 2022) but recent genetic studies have found that southern rockhopper penguins should be split into two sister taxa, southern and eastern rockhopper penguins (Banks et al., 2006; Frugone et al., 2021). We therefore separated the rockhopper penguins into three taxa (northern rockhopper penguins, southern rockhopper penguins and eastern rockhopper penguins). Furthermore, royal penguins are genetically similar to macaroni penguins, despite their clear phenotypic differences (Cole et al., 2019; Frugone et al., 2019), so we used this opportunity to also predict where royal penguins could forage during their post‐moult migration as currently there are no tracking data for this species for this stage of their annual cycle. Therefore, in this study, we included the royal penguin as part of the macaroni penguin group. All species breed on Subantarctic islands (Figure 1) apart from northern rockhopper penguins on Subtropical Amsterdam Island, St. Paul Island (Central Indian), Tristan, Nightingale, and Inaccessible Islands (Atlantic). Southern rockhoppers are found in the southwestern Atlantic Ocean around the southern tip of South America, and eastern rockhoppers are found in the southwest Indian and Pacific Oceans. Macaroni penguins are mostly restricted to oceanic islands across the Subantarctic, with small populations on islands off South America and on the Antarctic Peninsula. Royal penguins are confined to Macquarie Island. Northern rockhopper penguins are listed as Endangered, and sister taxa southern and eastern rockhopper species are listed as Vulnerable (IUCN, 2022). Macaroni penguins are classified Vulnerable (IUCN, 2022) and royal penguins are Near Threatened (IUCN, 2022). All taxa have historically experienced or are still experiencing decline in their population numbers (see Table S1 for details).

All statistical analyses were performed using R Statistical Software 4.1.0 (R Core Team, 2021).

### 2.2 Tracking data

This study estimated the post‐moult movements of penguins using light‐based geolocation (GLS) tags deployed between 2006 and 2020. Tracking data were assembled from 471 individual penguins: 223 southern rockhopper, 114 northern rockhopper, 81 eastern rockhopper and 75 macaroni penguins (see Table S2 for details on tracking years and sample sizes). Data are from 10 of the 21 major breeding sites including the Falkland Islands (Malvinas; United Nations, 2022), Isla de los Estados, Gough and Nightingale Islands (Tristan da Cunha), Amsterdam Island, Prince Edward Islands, Crozet Islands, Kerguelen Islands, Campbell Island and South Georgia (see Table S3 for Data Availability). GLS tags are miniaturized archival data tags with long battery lives, which make them currently the only suitable method to capture a full post‐moult migration while minimizing the burden on the animals (Bost et al., 2009). Tags were mounted on the birds' legs (during the breeding season or at the end of the moult, depending on the colony) using purpose‐designed bands (e.g. Ratcliffe, Crofts, et al., 2014; Ratcliffe, Takahashi, et al., 2014). The tags were recovered when the birds returned to their colonies to breed. As well as light level measurements, which can be used with local time to estimate latitude and longitude twice a day, the devices whenever possible also recorded sea surface temperature (SST). We analysed 485 full tracks (163, 433 location points in total) from 597 raw light files (raw light files that were faulty or with partial recordings were not included) using the *BAStag* and *SGAT* packages in R (S. Wotherspoon et al., 2013b; S. J. Wotherspoon et al., 2013a) with an ice mask around Antarctica. Where tags collected temperature data, a SST prior was used to constrain the birds' location based on Reynolds weekly SST maps (Lisovski et al., 2019; Reynolds et al., 2005).

Prior to statistical analyses, these tracks were then processed using a hierarchical state‐space model (SSM) using the *bsam* package (Jonsen et al., 2020). We did this to estimate locations at regular intervals (12 h) as well as provide movement parameters (trip duration, turning angles, maximum distances from the colony and travel speeds) used to simulate biologically meaningful tracks (see below).

### 2.3 Simulating tracks to estimate the random occurrence likelihood

We used a case–control design for habitat preference modelling of the observed tracking data, where environmental characteristics along the observed tracks were compared to those along a set of simulated tracks (Aarts et al., 2008). This is like a presence‐background design in general habitat preference modelling. Simulated tracks represent a set of locations where an animal would move with no habitat preference but with the same movement characteristics (identical dates, trip durations, maximum distances from the colony and travel speeds) of the observed tracks. They represent a set of background location estimates which represent the likelihood of random occurrence and that consider the geographic availability of cells to animals (Raymond et al., 2015). To do this, we simulated 50 tracks for each observed track using movement parameters from the SSM, with the *availability* package in R (Raymond et al., 2015; Reisinger et al., 2018). This allowed the habitat preference of the animals to be modelled using the environmental characteristics at the locations where the animals were observed and those at locations that they occurred randomly. The number of simulated tracks were chosen as a balance between adequate coverage of the random use of the marine environment and limiting the dataset size for computation (Hindell et al., 2020).

Simulated tracks were constrained to an ecologically realistic geographic space that is accessible to these taxa of *Eudyptes* penguins during their winter period. We have prior knowledge of broad‐scale habitat use (e.g. the birds do not visit tropical or high latitude areas) so including these would result in the avoidance of these regions dominating the habitat preference response. We therefore latitudinally constrained the simulated tracks to be between 30°S and the northern extent of the ice edge, defined here as <80% ice concentration. This prevented simulated tracks from unrealistically extending into tropical waters to the north or into areas which would be covered by winter sea ice in the south. Simulated tracks were also constrained by a land mask which prevented any tracks being located on land.

### 2.4 Environmental data

To characterize the bio‐physical conditions associated with utilized (observed) and random (simulated) locations, we used a suite of nine environmental covariates (Table 1). Environmental data were remotely sensed, or model estimated, and represented variables known to influence penguin distributions (Péron et al., 2012; Thiebot et al., 2013; Whitehead et al., 2016) or likely to influence the distribution of the prey (Pinkerton et al., 2020). Environmental data from *Copernicus Marine Service* (www.marine.copernicus.eu) were used (Table S4). The environmental data associated with the observed and simulated tracks were extracted using the *raster* package (Hijmans et al., 2015) by matching location and date at a 1° × 1° spatial resolution and daily time scale. Thereafter, to predict preferred habitats at a Southern Ocean wide scale in the habitat preference model step, mean climate data (climatologies) were compiled from the same environmental variables ranging from April to September (austral autumn and winter when these taxa of *Eudyptes* penguins migrate) spanning the length of time which the tracks covered (2006–2020).

**TABLE 1:** Summary of covariates used to model the at‐sea distribution of the five taxa of Eudyptes penguins in this study

Variable	Unit	Ecological significance
Sea surface height	m	Broad scale environmental features, proxy for different oceanic frontal zones, water mass properties
Sea surface height anomaly	m	Mesoscale oceanographic features, including mesoscale eddies
Bathymetry	m	Static features, water mass properties
Bathymetry gradient	°	Static features, seafloor slope
Sea surface temperature	°C	Water mass properties, represents areas of different prey and prey aggregations
Chlorophyll *a* concentration	mg m^−3^	Represents primary production, general positions of fronts, represents areas in certain cases of different prey and prey aggregations
Eddy kinetic energy	cm^2^ s^−2^	Dynamic oceanographic structures, including mesoscale eddies, jets, waves, and large‐scale motions
Mixed layer depth	m	Influences the depth of thermocline and prey fields from the surface: important for air‐breathing diving predators
Sea ice concentration > 80%	%	Used to constrain penguin tracks to north of the sea ice extent

### 2.5 Habitat preference models

Boosted regression trees (BRT) are a machine learning algorithm with a high predictive accuracy and abundant flexibility. In this study, we wanted to identify the environmental variables that are important to predict where these are for the global populations now and in the future. The predictive capability of BRT modelling is more robust compared to other models as the technique of boosting to combine large numbers of relatively simple tree models adaptively optimizes the predictive performance (Elith et al., 2006; Elith & Leathwick, 2017). Considering this and the ability of BRT modelling to deal with non‐linear relationships and correlated and interacting variables (common in ecological studies; Elith et al., 2006, 2008); we used BRT modelling to model penguin habitat preference as a function of their biophysical environment using the *gbm* package (Greenwell et al., 2019). BRT modelling was done by species. We used a learning rate of 0.01 and chose optimal number of trees (minimizing the prediction bias) using a *k*‐fold cross‐validation by individual (Elith & Leathwick, 2017). Robust estimates of the number of trees that can be used is important to avoid overfitting as the models need a degree of generality to predict successfully into unsampled areas (Schonlau, 2005). To increase the model accuracy, randomness was included using a bag refraction of 0.5 and tree complexity of 5. A Bernoulli family appropriate to the binary response variable (observed (1) vs. simulated (0)) was stipulated. The model outputs describe the probability of relative habitat preference. The habitat models were evaluated using number of trees, percentage of deviance, *R* ^2^ (the proportion of the variance for a dependent variable that's explained by an independent variable) and CV AUC (cross validation for area under the curve). Blocking validations were performed at the colony level (Roberts et al., 2017) to test the predictive performance of the species preferences models on individual colonies, with summary statistics exhibited with confusion matrices.

Although extensive, our tracking dataset lacked data for colonies of rockhopper on Macquarie and Auckland Islands and macaroni/royal penguins on Heard and McDonald Islands and Macquarie Island. To account for these data gaps, we used the habitat preference models to predict preferred habitat for each of the species across their entire ranges, including where no tracking data were available (see Table S1 for full list of breeding location used).

### 2.6 Accessibility

The data used to fit the habitat preference models (i.e. the observed and simulated tracks) represent habitat only and may include geographic areas that are not accessible to the animals (e.g. outside their foraging range; Aarts et al., 2008; Matthiopoulos, 2003). As penguins return to their breeding colonies at the end of the post‐moult migration, it was important to consider the accessibility of a location (given they cannot just keep swimming endlessly). Habitat preference models were therefore constrained using accessibility models (models of areas that the birds could feasibly access) to produce an index that reflected both the habitat preference of a given cell and how accessible that cell is to the birds (Hindell et al., 2020). Accessibility was modelled by using observed and simulated tracks as a function of the distance of the cell to the deployment colony. The number of locations were converted to a binary response (observed and simulated tracks vs. no observed and no simulated location). Then binomial models were fitted with a smooth, monotonic decreasing constraint as we assumed that accessibility decreases the further away from the colony. This additional modelling step was used to estimate which areas of geographic space were accessible to the birds by upweighting cells that were both preferred habitat and accessible and down‐weighting cells that were preferred habitat but not accessible. These outputs provide an estimate of the probability that animals from those colonies would be able to visit a given cell. This allows, for any colony, predictions to be made for the accessibility of each grid cell in the study region as well as its relative habitat suitability. An advantage of using separate models for accessibility and habitat suitability is that the predictor variables (and the shape of the response curves) can be different in the two models (current and predicted climate change models), allowing more ecologically realistic models (Hindell et al., 2020).

These accessibility models could be unweighted or weighted by colony size. The weighted models, which considered population sizes of colonies, up‐weighted cells in the vicinity of larger colonies and down‐weighted cells around smaller colonies thus giving an indication of density. Here, we focussed on unweighted predictions as we cannot know what population sizes will be at the end of the century (2071–2100), the period to which our climate change predictions are forecast, nor how these will be distributed across available breeding locations. However, the predictions based on habitat preference and population density based on current environmental conditions and population sizes are presented in Figure S3 for comparison.

### 2.7 Mapping

We used our model fits to predict habitat preference for each taxon and to allow comparisons across taxa. However, habitat preference predictions are not absolute estimates of probability and are therefore not directly comparable between species (Beyer et al., 2010). To allow for comparison between the five taxa, each prediction map was transformed by percentile to give a habitat importance score (Raymond et al., 2015). These final maps present areas of important habitat and are therefore comparable between species, depicting habitat represented on a scale from 0 (not important at all) to 100 (extremely important).

### 2.8 Redistribution of preferred habitat under climate change projections

To model climate change redistributions, we used two pathways of atmospheric greenhouse gas concentration: RCP4.5 and RCP8.5, which correspond to medium and high radiative forcing, respectively. We used phase five of the World Climate Research Programme's Coupled Model Inter‐comparison Project (CMIP5; https://www.wcrp‐climate.org/wgcm‐cmip/wgcm‐cmip5) output to allow our results to be directly comparable with predictions made for climate change redistributions of other predators in the Southern Ocean by Hindell et al. (2020) and Reisinger et al. (2022). Climate data were compiled using eight global CMIP5 climate models (ACCESS1.0, BCC‐CSM1.1, CanESM2, CMCC‐CM, EC‐EARTH, GISS‐E2‐H‐CC, MIROC‐ESM, and NorESM‐M; henceforth referred to as CMIP5 representations) considered to be the most suitable for Southern Ocean studies (Cavanagh et al., 2017). These projections provide an indication of the redistribution of preferred habitat under the assumption that colonies will not move, and habitat preferences will not change.

**FIGURE 2:**
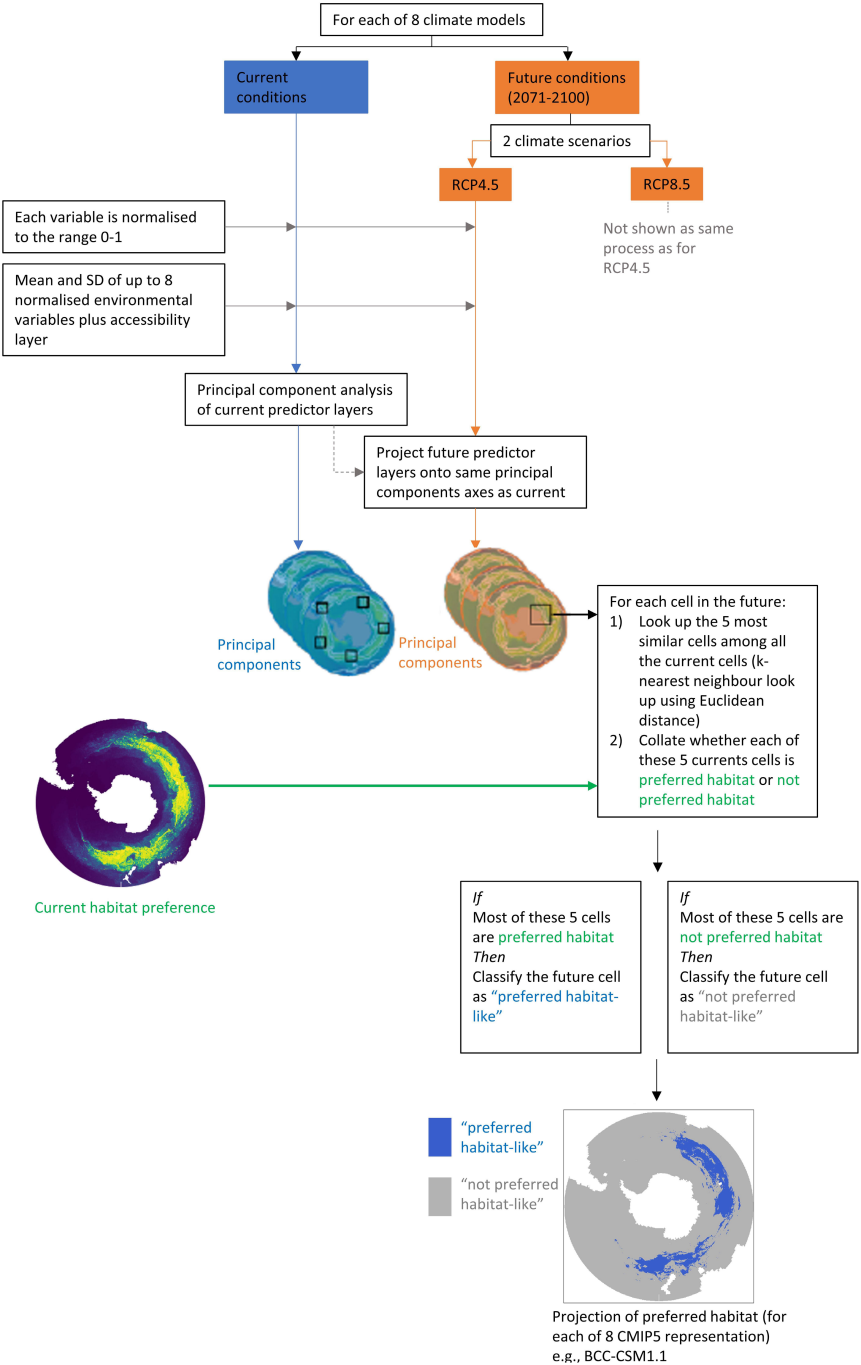
Methodology of the process used to make future predictions of the redistribution of preferred habitat under future climate change scenarios (adapted from Hindell et al., 2020).

In theory, it is possible to use hindcast CMIP5 data and future projections of CMIP5 data and accessibility to predict current preferred habitat and future projections of habitat preference. However, some predictor variables (Table 1) are not available from the CMIP5 data while others may have different properties due to the different temporal and spatial output. To produce biologically meaningful interactions for the current habitat preference models, we used satellite derived altimetry and reanalysis data (Table 1). Hindcast CMIP5 data are not as accurate as real time data and would produce less reliable current habitat preference model predictions. So, to predict future distributions of preferred habitat, we instead used a *k*‐nearest neighbour classifier approach (Figure 2). This matched the five most similar cells between the current preferred habitat and current‐climate grid, taking into account environmental variables and accessibility conditions. Projected habitat preference redistributions were then calculated by finding where those same current‐climate grid cells could move to, under future projected climate conditions using Euclidean distance. If the majority of those five cells were from current‐climate grid, the future‐climate grid cell was classified as “preferred habitat‐ like” and if not, then “not preferred habitat‐like” (Figure 2). This method allows comparison of output from multiple CMIP5 representations, each representing different suites of biophysical variables. Thereafter, the eight redistribution models could be combined into one generalized climate change projection per species and per RCP scenario.

We used a maximum of eight environmental variables per CMIP5 representation (Table S6), depending on the suite of variables available for that representation (i.e. not every CMIP5 representation contained all eight variables). Variables were normalized to range 0–1 prior to further analyses. As the resulting environmental variables showed high correlation, we used a principal components analysis to reduce the number of inter‐correlated predictor variables in the data set. The lowest number of principal components required to explain at least 95% of the variance was used.

To assess projected change in habitat area (reduction or increase) per species at a regional scale, we divided the Southern Ocean into the 5 regions using the *MEASOshapes* package (Brasier et al., 2019) into the Atlantic, Central Indian, East Indian, West Pacific and East Pacific (see Figure S5). We calculated the percentage change between the area of current habitat and the area of predicted habitat for the future (2071–2100) for each of the eight CMIP5 representations to account for the variability between them. We then plotted the degree of change per CMIP5 representation for each penguin species and each region where the species exists to compare the differences in habitat change between scenarios RCP4.5 and RCP8.5.

## 3 RESULTS

### 3.1 Current habitat preference

**FIGURE 3:**
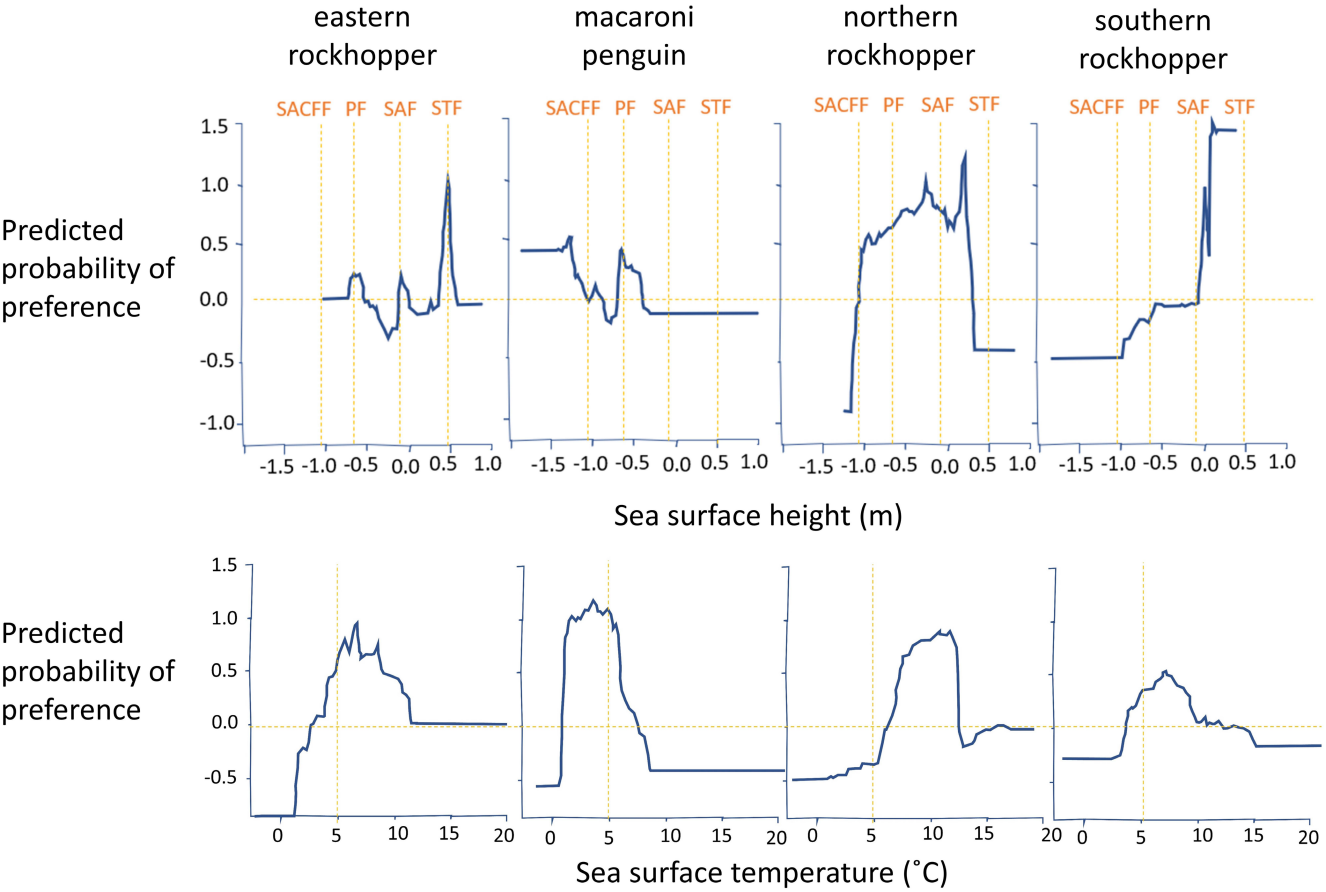
Habitat model response curves for sea surface height and sea surface temperature for the five focal *Eudyptes* penguin taxa: eastern rockhopper (*E. filholi*), macaroni (*E. chrysolophus*)/royal (*E. schlegeli*) penguins, northern rockhopper (*E. moseleyi*), and southern rockhopper (*E. chrysocome*). Positive predicted probability of preference suggests higher preference for those ranges of that environmental variable and negative predicted probability of preference suggests the animals actively do not select those ranges of the environmental variables. The higher the positive predicted probability of preference is, the more those ranges of the environmental variable are actively selected. Orange gridlines are for references: Horizontal lines are 0.0; vertical lines for sea surface heights are STF = 0.5 m (Subtropical Front), SAF = 0.128 (Subantarctic Front), PF = −0.634 (Polar Front) and SACCF = −1.09 (Subantarctic Circumpolar Current Front); and sea surface temperature is the 5°C point. Definitions for the front locations according to sea surface heights taken from Orsi et al. (1995) and Venables et al. (2012).

Model performance (AUC) ranged from 0.805 ± 0.005 to 0.934 ± 0.002 with a percentage of deviance explained 58.5% to 78.8% (Table 2). While predictor variable importance differed across the species, sea surface temperature (importance range of 11.8–80.4%, mean = 32.5%) and sea surface height (importance range of 17.1–25.5%, mean = 21.5%) were the most consistent predictors of habitat preference (Figure 3). Summary statistics from the blocking validations (Table S5) indicate lower predictive capacity for some species. The models performed best for Macaroni penguins and least well for northern rockhopper penguins reinforcing the need to interpret our predictions with caution.

**TABLE 2:** Statistics of model performance: Model performance statistics for eastern rockhopper (*E. filholi*), macaroni (*E. chrysolophus*)/royal (*E. schlegeli*) penguins, northern rockhopper (*E. moseleyi*), and southern rockhopper (*E. chrysocome*) with number of trees, percentage of deviance explained, *R* ^2^ (the proportion of the variance for a dependent variable that's explained by an independent variable) and CV AUC (cross validation for area under the curve)

Species	Number of trees	Percentage of deviance explained	*R* ^2^	CV AUC
Eastern rockhopper penguin	3500	71.9	0.534	0.805 ± 0.005
Macaroni/royal penguins	3200	78.8	0.778	0.934 ± 0.002
Northern rockhopper penguin	2000	67.3	0.562	0.87 ± 0.001
Southern rockhopper penguin	2800	58.5	0.482	0.822 ± 0.006

Important areas for eastern rockhopper penguins were characterized by waters between 1000–2000 m and 2800–3800 m deep (Figure S2), sea surface height levels between 0.4 and 0.6 m and sea surface temperature between 6 and 10°C (Figure 3). The single most important predictor of distribution for macaroni/royal penguins, contributing ~80% to the habitat model, was sea surface temperature. Macaroni/royal penguins showed the highest prevalence between sea surface temperature between 3–4.5°C (range of 2–6°C; Figure 3). The second most important predictor of suitable habitat for macaroni penguins was depth, that is, the preferential use of waters 3000–5000 m deep (Figure S2).

The two predictors that contributed ~50% to the model when predicting northern rockhopper penguin important habitat were sea surface temperature between 10 and 12°C and sea surface height between 0.15 and 0.25 m (Figure 3). The third most important predictor of suitable habitat for northern rockhopper penguins was sea surface height anomaly with a preference for positive anomalies. Important areas for southern rockhopper penguins generally were characterized by sea surface height of over 0.1 m, waters where mixed layer depth was between 0–50 m depth and between 250 and 300 m depth (Figure S2) and sea surface temperature that ranged from 6.5–9°C (Figure 3).

**FIGURE 4:**
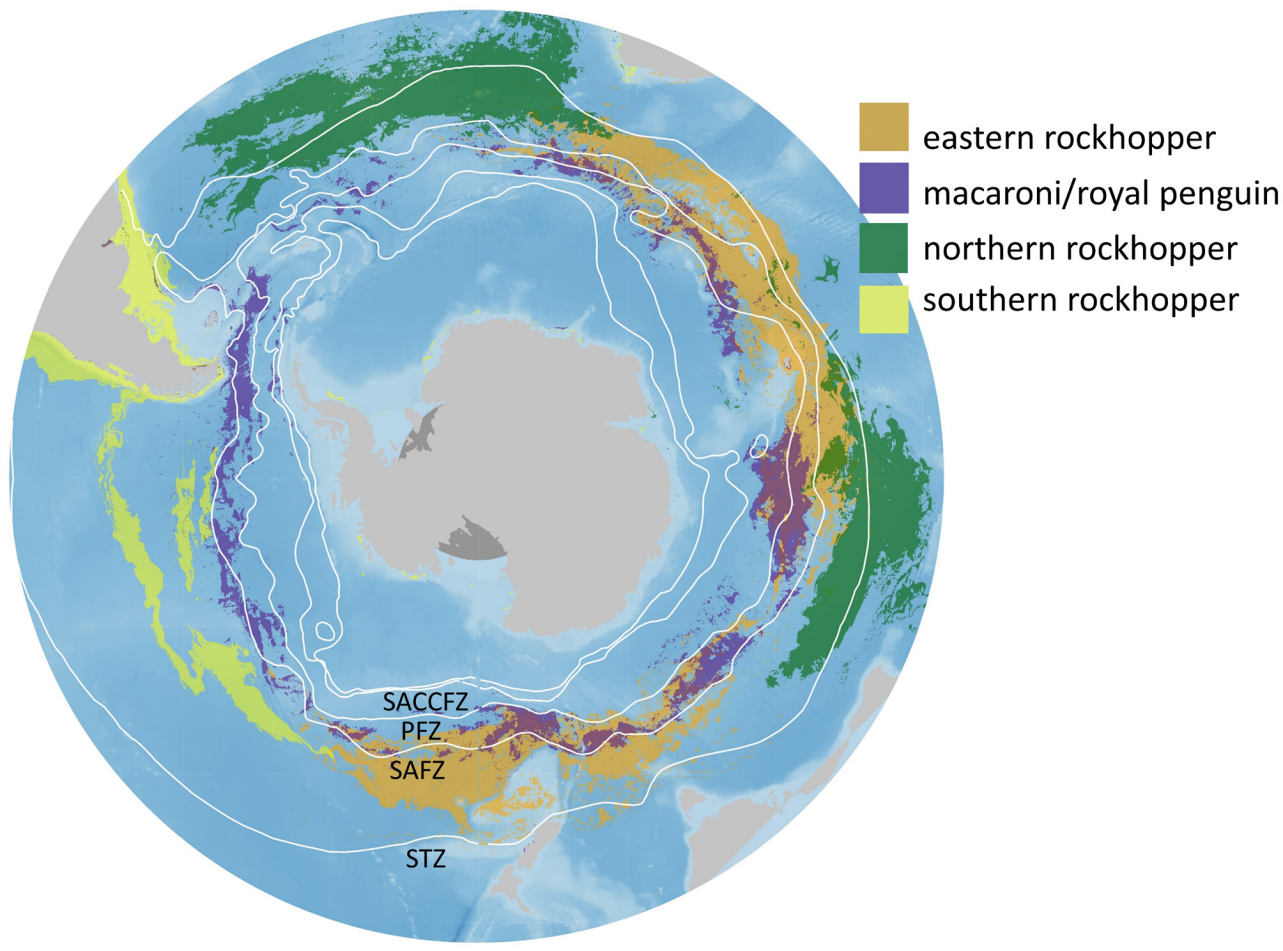
Relative habitat preferences, showing top 10 percentile, for five taxa of *Eudyptes* species: eastern rockhopper (*E. filholi*), macaroni (*E. chrysolophus*)/royal (*E. schlegeli*) penguins, northern rockhopper (*E. moseleyi*) and southern rockhopper (*E. chrysocome*) with the mean generalized major oceanographic frontal zone locations as defined by Orsi et al., 1995 (Southern Antarctic Circumpolar Frontal Zone = SACCFZ, Antarctic Polar Frontal Zone = PFZ, Subantarctic Frontal Zone = SAFZ, Subtropical Frontal Zone = STZ; using the Orsi Fronts). The purple layer is above the other colour layers.

Apparent spatial segregation in preferred habitats was noticeable between the five penguin taxa (Figure 4). Important eastern rockhopper penguin habitat occurred in regions encompassing the Australasian Subantarctic Islands and southern Indian Ocean, as well as the Kerguelen, Crozet and the Prince Edward Island groups. The areas highlighted were largely restricted to the Subantarctic Frontal Zone. Important habitat for macaroni/royal penguins highlighted a circumpolar distribution, largely within the Antarctic Polar Frontal Zone as well as the Southern Antarctic Circumpolar Frontal Zone. Preferred habitat for northern rockhopper penguins occurred north of the Subtropical Front and within the Subantarctic Frontal Zone. Important southern rockhopper penguin habitat was predicted along the coastline of Patagonia and Chile and into the Pacific Ocean, west of Chile (Figure 4). For this species, two distinct regions were predicted to be important: the South American continental shelf, used by birds from the Falkland Islands (Malvinas), and open ocean waters of the south‐east Pacific, used by birds from Beauchene and Staten Island/Isla de los Estados.

### 3.2 Climate change redistributions

**FIGURE 5:**
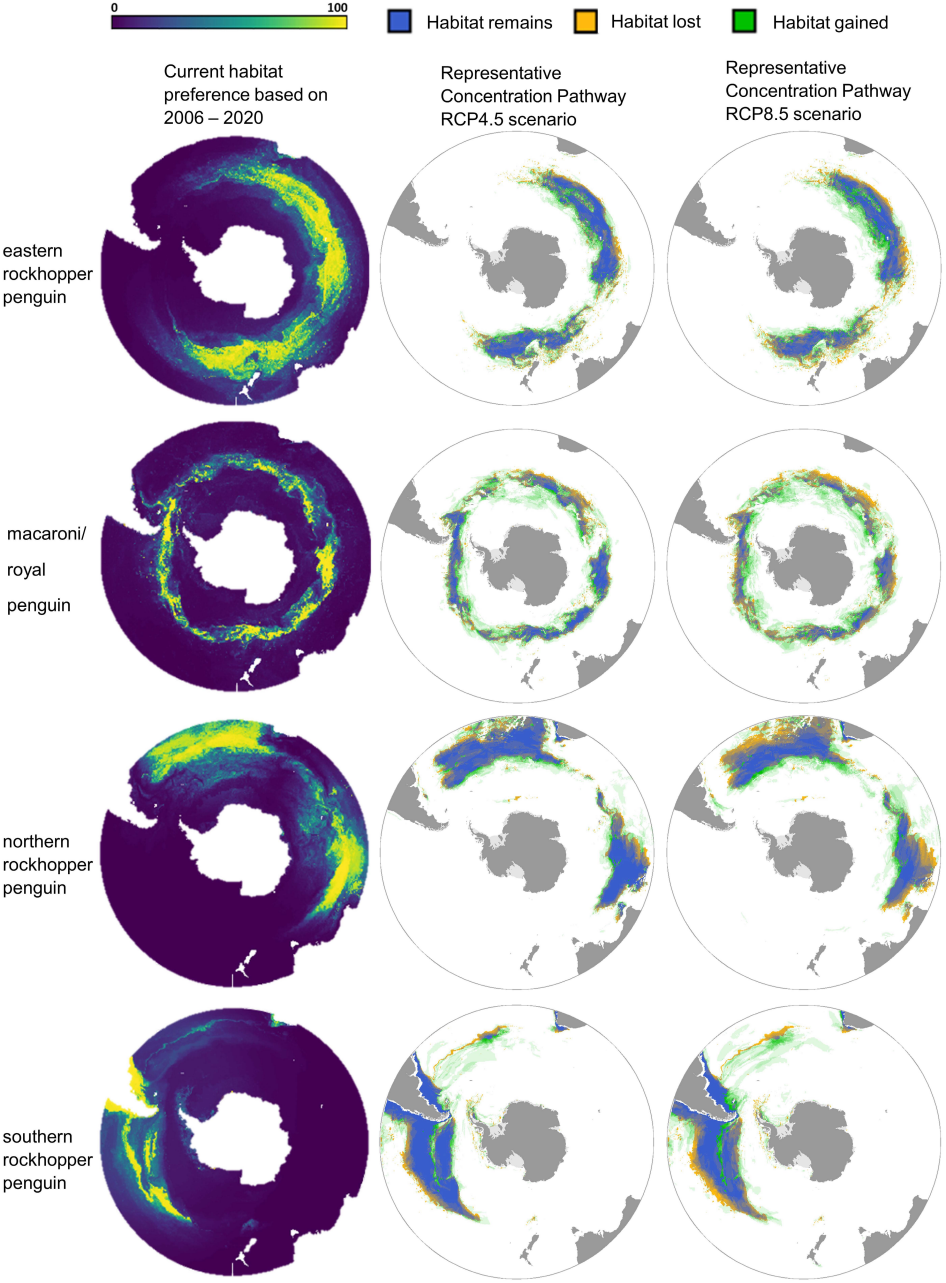
Changes in habitat preference for five taxa of *Eudyptes* penguin under climate change predicted conditions. The figures represent polar projections of current important habitat for: eastern rockhopper (*E. filholi*), macaroni (*E. chrysolophus*)/royal (*E. schlegeli*) penguins, northern rockhopper (*E. moseleyi*), and southern rockhopper (*E. chrysocome*), and predicted habitat changes under Representative Concentration Pathway RCP4.5 (which assumes global temperatures increasing by 1.1–2.6°C and a mean sea level rise of 0.47 m, by the year 2100) and RCP8.5 (which assumes global temperatures increasing by 3.0–12.6°C and a mean sea level rise of 0.62 m, by the year 2100 (Sabine, 2014), which correspond to medium and high radiative forcing, respectively. RCP4.5 and RCP8.5 scenarios depict: Habitat that could remain important (blue), habitat that could be potentially gained (green) and habitat that could be lost (orange).

**FIGURE 6:**
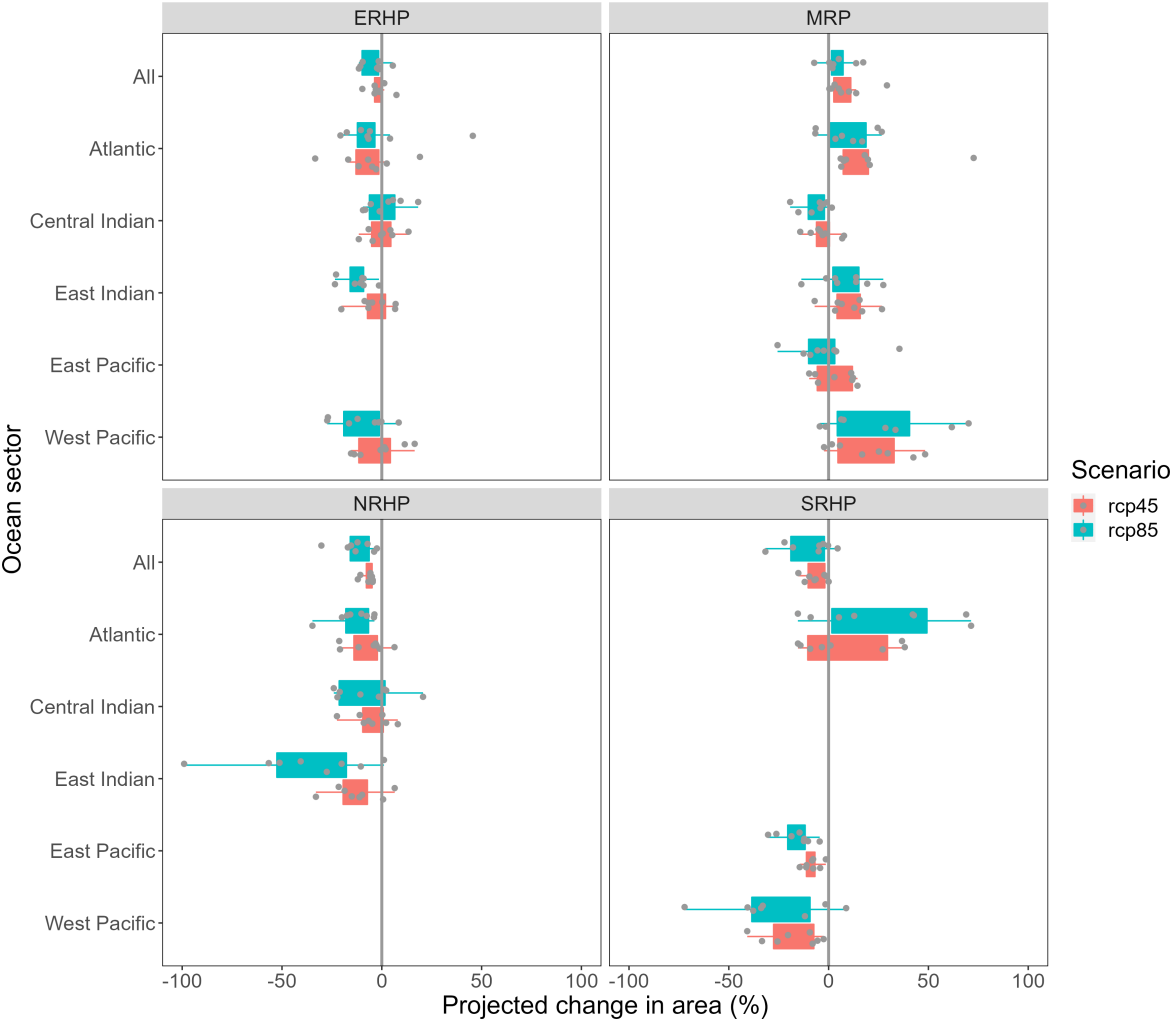
Percentage change in habitat area per region (using the Marine Ecosystem Assessment for the Southern Ocean defined regions) for the eight different climate representation (ACCESS1.0, BCC‐CSM1.1, CanESM2, CMCC‐CM, EC‐EARTH, GISS‐E2‐H‐CC, MIROC‐ESM, and NorESM‐M: grey points) for eastern rockhopper penguins (ERHP), macaroni/royal penguins (MRP), northern rockhopper penguins (NRHP) and southern rockhopper penguins (SRHP) under Representative Concentration Pathways RCP4.5 (red) and RCP8.5 (blue) scenarios. Percentage change for the eight climate change representations is shown by the dots. The boxes indicate the 25th–75th percentiles.

Broadly, for all species and across both scenarios (RCP4.5 and RCP8.5), preferred habitat was predicted to move poleward, with habitat being lost in the northern limits of the species' ranges and gained in the southern area of their ranges (Figure 5). For all rockhopper species, our results predicted a greater average loss of habitat for scenario RCP8.5 than scenario RCP4.5 (Figure 5). Overall, there was a projected net loss of preferred habitat for all three rockhopper species (Figure 6). Preferred habitat for eastern rockhoppers showed a mean areal reduction of −1.7% for RCP4.5 or by −4.2% under RCP8.5 (Figure 6). Northern and southern rockhopper penguins were projected to undergo similar losses of preferred habitat area under RCP4.5 (−6.9% and −6.8%, respectively). However, this predicted loss intensified under RCP8.5: −12.7% and −9.9% for northern and southern rockhopper penguins, respectively. In contrast to the three rockhopper species, our findings suggested an extension in macaroni/royal penguin preferred habitat, showing respective gains of 8.7% and 4.4% under RCP4.5 and RCP8.5.

Regionally, preferred habitat was projected to decrease for eastern rockhopper penguins in the East Indian (−4.2% and −12.5% for RCP4.5 and RCP8.5 scenarios respectively) and West Pacific regions (−1.1% and −9.9%) but with little change in the Central Indian (+0.03% and +1.5%). In contrast, regional macaroni/royal penguins preferred habitat showed projected increases in the Atlantic (+19.9% and +9.6%), East Pacific (+3.8%), East Indian (+9.9% and +8.5%) and West Pacific (+20.9% and +25.2%), with the only reductions occurring in the Central Indian region (−2.8% and −6.6%) and East Pacific under RCP8.5 (−1.6%). Northern rockhopper penguins were projected to experience a decrease in preferred habitat across their full range: Atlantic region (−7.3% and −14.2%), Central Indian (−5.4% and −6.9%) and East Indian (−12.7% and −38.1%). Our results also indicated that preferred habitat for southern rockhoppers may decline in the East Pacific (−8.3% to −16.1%) but with a notable increase in the Atlantic region (+7.6% and +27.4% under RCP4.5 and RCP8.5 respectively).

Areas of notable preferred habitat change (increase or decrease >20%) were an increase in area for macaroni/royal penguins in the Atlantic and southern rockhopper penguins in the East Pacific (decrease) and the Atlantic regions (increase). Our results indicate that warming in the Atlantic region could increase preferred habitat for macaroni penguins at suitable breeding sites including Bouvet, South Georgia, South Orkney, South Sandwich, South Shetland, and the Antarctic Peninsula. Other regional changes of interest (>10%) were a decline in habitat for eastern rockhopper penguins in the East Indian and West Pacific region (populations that exist on the Australasian Subantarctic Islands), an increase for macaroni/royal penguins in the East Indian (reflecting the royal population at Macquarie Island) and decline in habitat for northern rockhopper penguins in the Atlantic region and southern rockhoppers habitat in the East Pacific.

## 4 DISCUSSION

Here, we identified preferred habitat during the post‐moult migration of five *Eudyptes* penguin taxa from a large tracking dataset from 21 populations across almost all major breeding locations and projected how their extent and distribution could change under the RCP4.5 and RCP8.5 climate scenarios. The five taxa of penguin may experience a general poleward redistribution of their preferred habitat, but with contrasting effects in the (i) change in total area of preferred habitat under climate change (ii) according to geographic region and (iii) the species (macaroni/royal vs. rockhopper populations). Our results indicated that the less severe RCP4.5 would lead to less overall habitat loss by the end of the century than the more severe RCP8.5 scenario. These findings are in agreement with other studies on climate change‐induced redistributions that show current habitat preferences of marine animals shifting poleward (Clairbaux et al., 2021; Hindell et al., 2020; Quillfeldt et al., 2010), but at rates differing across regions and among populations (Reisinger et al., 2022).

### 4.1 Current habitat preferences

Post‐moult habitat preferences for all species largely coincided with the distribution of dominant frontal zones, particularly within open ocean habitat south of the Subtropical Front (STF; Figure 4). For rockhopper penguins, foraging was generally located within the Subantarctic/Antarctic inter‐frontal zone, whereas macaroni/royal penguins tended to prefer the Polar frontal zone. Latitudinal separation in the use of different inter‐frontal zones by penguin species may reflect differences in prey preference but is also seen by the breeding locations of macaroni/royal penguins, which generally breed at higher latitudes (Figure 1). Resource partitioning mechanisms in areas where species overlap may include vertical spatial partitioning and temporal partitioning such as allochrony (Green et al., 2022; Ratcliffe, Crofts, et al., 2014; Thiebot et al., 2013; Whitehead et al., 2016) to reduce competition.

These findings support previous work documenting strong preferences for frontal zones by marine predators (Bost et al., 2009; Green et al., 2022; Thiebot et al., 2013). Fronts act as biogeographic barriers defining the distributions of species (Atkinson et al., 2012; Pakhomov et al., 1996), and influencing the distribution of prey. For example, seabird assemblages in the Southern Ocean, including the *Eudyptes* guild, have previously been associated with specific frontal zones, due to the inferred distribution of prey structured by oceanographic fronts (Bost et al., 2009; Thiebot et al., 2012, 2013; Thiebot, Lescroël, et al., 2011). The regions that are important for these taxa of *Eudyptes* penguins occur largely between the south Antarctic Circumpolar Current Front and the STF and are consistent with the distribution of high concentrations of zooplankton (Pinkerton et al., 2020) including euphausiids which are the dominant prey for *Eudyptes* penguins (Cherel et al., 2007; Rey et al., 2005).

Subantarctic waters (predominantly used by rockhopper species) are generally dominated by smaller euphausiids such as *Euphasia vallentini*, whereas larger species such as *E. superba* (Antarctic krill), *E. frigida* and *E. triacantha* occupy waters south of the Polar Front (Endo et al., 2008), highlighting that these two frontal zones are characterized by different prey size assemblages. The distribution of euphausiids is particularly important for rockhopper species that typically occupy a lower trophic level (Dehnhard et al., 2011). In contrast, macaroni penguins generally consume larger, higher trophic level prey items than rockhoppers including a higher proportion of myctophid mesopelagic fishes (Cherel et al., 2007; Cooper et al., 1990).

Overall, the importance of frontal zones in controlling biogeography could have considerable implications for prey accessibility for penguins under climate‐mediated change. Habitat use by migrating penguins is determined by swimming speed capabilities, currents that assist/hinder movements as well as the profitability of oceanic feeding areas (Bon et al., 2015; Della Penna, 2016). Biophysical conditions in the Southern Ocean are changing rapidly (Constable et al., 2014), and whereas cues used by foraging penguins will likely to remain unchanged in the short term (according to generation times of macaroni penguin 8–15 years and rockhopper species 6–10 years), the biophysical features of the ocean and associated locations and availability of their prey may not. Currently, sea surface temperature is changing with the implication that preferred prey species may be pushed farther south or deeper into the water column to follow the cooler waters (Constable et al., 2014; Freer et al., 2019; Veytia et al., 2020). Results outlined in this study suggest that similar shifts in the distribution of suitable winter foraging habitat could occur for these taxa of *Eudyptes* penguins as well.

### 4.2 Potential consequences of habitat redistribution for penguin post‐moult foraging ecology

Regionally, interactions of different climate forcings will act on the chemistry and biology of primary and secondary production, with implications for regional food web structure (Sydeman et al., 2015; Trathan et al., 2007). Populations of marine predators from a particular species that breed in different regions may occupy different niches depending on the regionally dominant food web. The blocking cross‐validations suggest that this is the case for the eastern and northern rockhopper species due to the low transferability of models between certain populations/species (Torres et al., 2015). Habitat preferences differ between colonies as there are likely different prey types available, each with their own habitat preferences. Because of this, marine predator responses to climate‐driven change could differ across populations based on variation in prey composition. For example, macaroni penguins consume Antarctic krill as their main prey at South Georgia in the Atlantic (Waluda et al., 2012), while their diet in the Central Indian Ocean is dominated by the crustaceans *Euphausia vallentini*, *Thyssanoesa vicina* and *Themisto gaudichaudii*, followed by myctophid fish and cephalopods (Cherel et al., 2007; Crawford et al., 2003). At Macquarie Island in the East Indian (MEASO‐defined region), myctophid fish *Krefftichthys anderssoni* dominate the diet of royal penguins, followed by *Euphausia vallentini* (Hull, 1999). These studies show that some species may be able to respond flexibly to changes in the availability of preferred prey. Therefore, while our models predict large‐scale redistributions of *Eudyptes* habitat, they do not consider the potential for the penguins to shift their diet towards novel prey items that may become numerically dominant (e.g. gentoo penguins *Pygoscelis papua*; Carpenter‐Kling et al., 2017).

While our study measured preferred habitat, it did not consider the full available habitat for each species. As such, while we have calculated change in preferred habitat extent, the species may still have access to other habitats within their physiological and morphological constraints within reach of their colonies, both currently and in the future (Pütz et al., 2021). Differences in diet and foraging strategies (e.g. increase in diving depth) within a species as well as individual specialization may buffer populations against changes in habitat (such as southern elephant seals *Mirounga leonina* from Macquarie Island: Hindell et al., 2017). For example, changes in regional habitat distributions could have contrasting effects on different southern rockhopper penguin populations. Reductions in preferred habitat in the East Pacific for southern rockhopper penguin may negatively affect populations that forage in these regions, such as those breeding on Staten Island/Isla de los Estados (Argentina) and Isla Noir (Chile; Oehler et al., 2018). Whereas populations breeding on the Falkland Islands (Malvinas) and smaller populations breeding along the Patagonian coast, which constitute about 40% of the population, could experience a large increase in preferred habitat. Southern rockhopper penguins from islands in the Atlantic region show a range of foraging strategies. Some colonies forage along the continental shelf and others forage in pelagic waters (Pütz et al., 2006; Ratcliffe, Crofts, et al., 2014; Thiebot et al., 2015). Both these areas (pelagic and shelf) coincide with the areas of increased preferred habitat.

Many studies that have investigated how penguin species could respond to a changing climate have focussed on Antarctic breeding, ice‐obligate species (however see, Cristofari et al., 2018; Péron et al., 2012). Like Antarctic penguins, changes for *Eudyptes* penguins relate to trophic mediated changes cascading from climate forcing. Antarctic penguins have adapted to sea ice conditions (Forcada et al., 2006) and are influenced by the availability of Antarctic krill and the effects of krill harvesting in Antarctic waters. In contrast, *Eudyptes* penguins are not ice dependent, have a more generalist diet and consume prey species that are generally not commercially valuable. All five taxa of *Eudyptes* in this study perform post‐moult migration round trips of ~10,000 km and cover a large area during the 4–6 months they are at sea. Given their generalist diet and ability to travel large distances, it could be expected that *Eudyptes* would be better equipped to adjust to environmentally driven changes in the distribution of suitable habitat. However, while regions used by these penguins are vast, key foraging areas used by wide ranging oceanic predators may represent only a small spatial subset of their overall range (Schofield et al., 2010). Areas important for foraging penguins are likely linked to the timing of the annual cycle (Moore et al., 1999), and the spatio‐temporal distribution of marine productivity and prey. For example, habitats used by penguins for hyperphagia prior to breeding and moulting are probably disproportionately important and are likely to be relatively close to breeding sites (Thiebot, Authier, et al., 2014; Thiebot, Cherel, et al., 2014). Changes in the extent and distribution of such habitats progressively away from their breeding colonies could reduce capacity for attaining sufficient condition to survive these energetically costly periods (Dehnhard et al., 2013).

Adjusting to the predicted changes could be also possible for penguins through the establishment of new colonies at sites closer to suitable overwintering habitat. However, data on natal dispersal of juvenile penguins in relation to climate change is currently lacking, and as adults show a high level of philopatry, they have a low emigration rate (Cristofari et al., 2015; Orgeret et al., 2022). Therefore, it is unlikely that changes in preferred habitat in the Southern Ocean would lead to the redistribution of adult birds by emigration, but rather a decrease in populations at sites that become unfavourable and growth of populations at sites that become more favourable. Where established colonies already exist on islands in the Atlantic and on the Antarctic Peninsula, the increase of preferred habitat to this region could lead to the increase in macaroni penguin populations (as it has for gentoo penguins; Forcada et al., 2006).

### 4.3 Uncertainties in projected future habitat distributions

Our results highlight sea surface temperature and sea surface height as being the most important predictors of foraging distributions for these species. Together, these variables may reflect broad water masses indicative of preferred prey fields. However, it is possible that these variables simply reflect the broad geographic space indicative of post‐moult migration, rather than key characteristics of their foraging habitat. This could have implications when considering how foraging habitat could change in future. In considering the circumpolar average, the Polar Front was reported to have already shifted by 60 km south since 1992 and was expected to continue to do so (Kim & Orsi, 2014; Sokolov & Rintoul, 2009). More recent work has led to the consensus that climate change models do not show a systematic shift in front locations (Chapman et al., 2020; Meijers et al., 2019), but that there is observed poleward warming of the frontal zones (Chapman et al., 2020). The suite of CMIP5 models chosen here all showed strong agreement towards a southward redistribution of penguin habitat. As sea surface temperature may indeed be the most important indicator of penguin foraging, as shown in our study as well (Cristofari et al., 2018; Le Bohec et al., 2008; Morrison et al., 2015; Pütz et al., 2018; Rey et al., 2007), the implications for poleward warming of the frontal zones may be significant enough to have negative effects on widely dispersive penguins. Sea surface temperature affects the locations of foraging for penguins and even short‐term poleward shifts of isotherms have resulted in decreases in survival rates and breeding success for penguins (associated with increased energy expenditure when foraging for food; Le Bohec et al., 2008; Trucchi et al., 2019).

Some areas of notable preferred habitat change were found that would be on the extreme edges of species ranges and unlikely to be used by the penguins. These were the notable decrease in preferred habitat for northern rockhopper penguins in the East Indian and southern rockhopper penguins in the West Pacific and increase for macaroni penguins in the West Pacific. Also, at least in the case of the rockhopper species, these areas are relatively small (see Figure S6). Thus, these specific changes, though they seem large in intensity, would likely have only minimal effects to the species.

### 4.4 Implications and conclusions

While this study does not measure the effects of changing extent of preferred habitat on population numbers, our results, at least in some regions, do seem to coincide with some population trends. Vagrant macaroni penguins have been seen increasingly further south on ice free islands off the Antarctic peninsula (Golubev, 2016; Gorman et al., 2010), a region predicted in our results to have an increase in preferred habitat for this species. Similarly, populations that have experienced severe declines are in regions predicted by this study to undergo reductions in preferred habitat (such as eastern rockhopper penguins from the New Zealand Subantarctic Islands and northern rockhopper penguins in the Atlantic, both of which have experienced <94% declines since 1940s; Cunningham & Moors, 1994; Cuthbert et al., 2009). Notably, while our models predict an increase in preferred habitat for populations from the Falklands, this is not reflected in population trends as long‐term declines have taken place from the 1930s to the early 2000s (Baylis et al., 2013; Pütz et al., 2003).

Our models predict that in some cases, shifts south in preferred habitat may occur by the end of the century, forcing some populations to possibly travel farther and spend more energy and time searching and foraging for food. Our habitat predictions only include habitats that are accessible to the penguins currently (according to the calculated distances they may feasibly travel from their colonies). Therefore, the increased habitat in the south of the penguins' ranges is within reach but may be at the upper limit of their energetics to reach.

Ongoing changes in preferred habitat across the Southern Ocean have the potential to result in large scale redistributions of populations like those that took place during the Last Glacial Maximum (*c*. 19.5–16 kya) after a significant climate warming (Cole et al., 2019) and the resultant overall decline in marine productivity (Vianna et al., 2020). Implications of our results could be that by the end of the century, some populations of the taxa of *Eudyptes* penguin in this study may decrease to a stable norm of lower numbers and some small existing colonies may start to increase (i.e. the relative importance of colony sites may alter). Our results reiterate previous work showing that responses to environmental change vary across species and between regions. Thus, we emphasize the need for species‐specific and region‐specific management and conservation against a backdrop of a rapidly altering marine environment. Finally, our results support those of Gervais et al. (2021), who argued that management needs to be adapted concurrently with changing conditions and should not wait for climate change impact research to justify action.

## FUNDING INFORMATION

This study forms part of a PhD funded by a British Ornithologists' Union John & Pat Warham Studentship from a bequest left to the BOU by the late John and Pat Warham for the study of sphenisciform and procellariiform seabirds by a student from Commonwealth countries.

## CONFLICT OF INTEREST

The authors declare that there is no conflict of interest.

## Supporting information


Appendix S1

## Data Availability

Current environmental data are available through *Copernicus Marine Service* (www.marine.copernicus.eu). CMIP5 data are available from phase five of the Coupled Model Intercomparison Project (CMIP5) of the World Climate Research Programme. Penguin tracking data are available through the Birdlife Repository (www.seabirdtracking.org; see Table S3 for full list of metadata and data repository links).
