# Supplementary material for: Potential for redistribution of post‐moult habitat for Eudyptes penguins in the Southern Ocean under future climate conditions
Source: Glob Chang Biol. 2022 Nov 9;29(3):648–67. doi: 10.1111/gcb.16500 (PMC10099906; doi:10.1111/gcb.16500)
Supplement: Supplementary file 1 — Appendix S1 [file GCB-29-648-s001.docx]

**Potential for redistribution of post-moult habitat for *Eudyptes* penguins in the Southern Ocean under future climate conditions**

Cara-Paige Green^1*^, David B. Green^1,2^, Norman Ratcliffe^3^, David Thompson^4^, Mary-Anne Lea^1,2^, Alastair M. M. Baylis^5,6^, Alexander L. Bond^7,8^, Charles-André Bost^9^, Sarah Crofts^10^, Richard J. Cuthbert^11,12^, Jacob González-Solís^13^, Kyle W. Morrison^4^, Maud Poisblau^14^, Klemens Pütz^15^, Andrea Raya Rey^16^, Peter G. Ryan^17^, Paul M. Sagar^4^, Antje Steinfurth^11^, Jean-Baptiste Thiebot^18^, Megan Tierney^5,19^, T. Otto Whitehead^17^, Simon Wotherspoon^20^, Mark A. Hindell^1,2^

^1^ Institute for Marine and Antarctic Studies, University of Tasmania, Hobart, Tasmania, Australia

^2^ ARC Australian Centre for Excellence in Antarctic Science, University of Tasmania, Institute for Marine and Antarctic Studies, Tasmania, Australia

^3^ British Antarctic Survey, Cambridge, UK

^4^ National Institute of Water and Atmospheric Research Ltd., 301 Evans Bay Parade, Hataitai, Wellington 6021, New Zealand

^5^ South Atlantic Environmental Research Institute, Stanley, Falkland Islands

^6^ Macquarie University, Sydney, NSW, Australia

^7^ RSPB Centre for Conservation Science, Royal Society for the Protection of Birds, The Lodge, Sandy, Bedfordshire, SG19 2DL, UK

^8^ Bird Group, Natural History Museum, Akeman Street, Tring, Hertfordshire, HP23 6AP, UK

^9^ Centre d’Etudes Biologiques de Chizé, UMR7372 CNRS-La Rochelle Université, 405 Route de Prissé La Charrière, 79360 Villiers en Bois, France

^10^ Falklands Conservation, Stanley, Falkland (Malvinas) Islands

^11^ Royal Society for the Protection of Birds, Centre for Conservation Science, David Attenborough Building, Pembroke Street, Cambridge, CB2 3QZ, UK

^12^ World Land Trust, Blyth House, Bridge Street, Halesworth, Suffolk, IP19 8AB, UK

^13^ Institut de Recerca de la Biodiversitat (IRBio) and Departament de Biologia Evolutiva, Ecologia i Ciències Ambientals, Universitat de Barcelona, Av Diagonal 643, Barcelona 08028, Spain

^14^ Behavioural Ecology and Ecophysiology Group, Department of Biology, University of Antwerp, Universiteitsplein 1, 2610, Wilrijk, Belgium

^15^ Antarctic Research Trust, Am Oste-Hamme-Kanal 10, 27432 Bremervörde, Germany

^16^ CADIC-CONICET, WCS Representación Argentina, Ushuaia, Tierra del Fuego, Argentina

^17^ FitzPatrick Institute of African Ornithology, DST-NRF Centre of Excellence, University of Cape Town, Rondebosch 7701, South Africa

^18^ National Institute of Water and Atmospheric Research Ltd., 10 Kyle Street, Riccarton, Christchurch 8011, New Zealand^18^ Graduate School of Fisheries Sciences, Hokkaido University, 3-1-1 Minato-cho, Hakodate, Hokkaido 041-8611, Japan

^19^ Joint Nature Conservation Committee, Peterborough, PE1 1JY United Kingdom

^20^ Australian Antarctic Division, Department of Agriculture, Water and the Environment, Kingston, Tasmania, Australia

Table S1: Distribution and Population Sizes for four taxa of Eudyptes penguin, southern rockhopper (*E. chrysocome*), northern rockhopper (*E. moseleyi*), eastern rockhopper (*E. filholi*) and macaroni (*E. chrysolophus*)/royal (*E. schlegeli*) penguins.

| **Breeding site** | **Co-ordinates**  **(°S; °W/E)** | **Past population estimates  from Cooper et al., 1990** | **Latest information  on population sizes (number of pairs)** | **Source of latest  population  numbers** |
| --- | --- | --- | --- | --- |
| **southern Rockhopper** |  |  |  |  |
| Falklands (Malvinas) | 51.79˚ S, 59.52˚ W | 2,500,000 | 320,000 | Baylis, Wolfaardt, Crofts, Pistorius, & Ratcliffe, 2013 |
| Isla Pingüino | 47.88˚ S, 65.89˚ W | 15,000 | 1,200 | Gandini, Millones, Morgenthaler, & Frere, 2017 |
| Isla de los Estados | 55.98˚ S, 67.26˚ W |  | 127,000 | Raya Rey, Rosciano, Liljesthröm, Sáenz Samaniego, & Schiavini, 2014 |
| Isla Noir | 55.43˚ S, 68.44˚ W |  | 158,200 | Oehler et al., 2008 |
| Diego Ramirez | 56.48˚ S, 68.73˚ W | 60,000 | 132,720 | Kirkwood et al., 2007 |
| Ildefonso | 55.75˚ S, 69.43˚ W |  | 86,400 | Kirkwood et al., 2007 |
|  |  |  |  |  |
| Total |  | 2,575,000 | 711,220 |  |
| **northern Rockhopper** |  |  |  |  |
| Tristian du Cunha | 37.1˚ S, 12.27˚ W | 140,000 | 3,584 | BirdLife International., 2020; Cuthbert et al., 2009 |
| Gough | 40.32˚ S, 9.93˚ W | 144,000 | 64,700 | BirdLife International., 2020; Cuthbert et al., 2009 |
| Middle/Alex | 37.4˚ S, 12.48˚ W | 100,000 | 62,791 | BirdLife International., 2020; Cuthbert et al., 2009 |
| Inaccessible | 37.3˚ S, 12.67˚ W |  | 33,867 | BirdLife International., 2020; Cuthbert et al., 2009 |
| Nightingale | 37.42˚ S, 12.47˚ W |  | 20,432 | BirdLife International., 2020; Cuthbert et al., 2009 |
| Amsterdam and St Paul | 37.83˚ S, 77.55˚ W | 1,300 | 8,100 | Barbraud et al., 2020 |
| Pointe d’Entrecasteaux | 38.72˚ S, 77.51˚ W |  | 12,161 | Barbraud et al., 2020 |
|  |  |  |  |  |
| Total |  | 385,300 | 205,635 |  |
| **eastern Rockhopper** |  |  |  |  |
| Prince Edward | 46.52˚ S, 37.45˚ E |  | 38,000 | Crawford et al., 2009 |
| Marion Island | 46.90˚ S, 37.75˚ E | 173,000 | 42,000 | Crawford et al., 2009 |
| Crozet Islands | 46.41˚ S, 51.98˚ E | 158,000 | 152,800 | Barbraud et al. 2020 |
| Kerguelen | 49.33˚ S, 70.33˚ E | 85,000 | 85,000 | Barbraud et al. 2020 |
| Heard and McDonald | 53.08˚ S,  73.5˚ E | 10,000 | 1010 | Woehler & Green, 1992 |
| Macquarie | 54.5˚ S,  158.9˚ E | 100,000 | 37,500 (range is 12,000 – 50,000) | Birdlife, 2018 |
| Auckland | 36.84˚ S, 174.76˚ E | 7,500 | unknown |  |
| Antipodes | 49.66˚ S, 178.76˚ E | 50,000 | 2,475 | Hiscock & Chilvers, 2014 |
| Campbell | 52.54˚ S, 169.14˚ E | 61,000 | 33,000 | Morrison et al., 2015 |
|  |  |  |  |  |
| Total |  | 644,500 | 391,785 |  |
| **macaroni/royal penguin** |  |  |  |  |
| Patagonian shelf region | 52.77˚ S, 74.62˚ W | 15,000 | 25,000 | Crossin, Trathan, & Crawford, 2013; Oehler et al., 2008 |
| Ildefonso | 55.75˚ S, 69.43˚ W |  | 5,660 | Kirkwood et al., 2007 |
| Isla Noir | 55.43˚ S, 68.44˚ W |  | 3,470 | Oehler et al., 2008 |
| Diego Ramirez | 56.48˚ S, 68.73˚ W | 10,000 | 15,600 | Kirkwood et al., 2007 |
| Falklands/Malvinas | 51.79˚ S, 59.52˚ W |  | 1,000 | Birdlife, 2018 |
| Antarctic Peninsula |  |  | <50 | Petry, Valls, Petersen, Finger, & Krüger, 2018; Schrimpf, Che-Castaldo, & Lynch, 2020 |
| South Georgia and South Sandwich | 54.28˚ S, 36.5˚ W | 5,410,000 | 2,057,234 | Horswill, 2015; Trathan, Ratcliffe, & Masden, 2012 |
| South Shetlands | 62.02˚ S, 58.21˚ W | 7000 | 1700 | Strycker et al., 2021 |
| Bouvet | 54.41˚ S, 3.36˚ E | 100,000 | 1200 | Niemandt et al., 2015 |
| Prince Edward | 46.52˚ S, 37.45˚ E |  | 12,000 | Crawford et al., 2009 |
| Marion | 46.90˚ S, 37.75˚ E | 422,000 | 290,000 | Crawford et al., 2009 |
| Crozet | 46.41˚ S, 51.98˚ E | 1,885,000 | 2,200,000 | Barbraud et al., 2020 |
| Kerguelen | 49.33˚ S, 70.33˚ E | 1,812,000 | 1,800,000 | Barbraud et al., 2020 |
| Heard and McDonald | 53.08˚ S, 73.5˚ E | 2,000,000 | 1,000,000 | Birdlife, 2018; Crossin, Trathan, & Crawford, 2013 |
| Macquarie | 54.5˚ S, 158.9˚ E | 850,000 | 750,000 | Salton, Kliska, Carmichael, & Alderman, 2019 |
|  |  |  |  |  |
| Total |  | 12,511,000 | 7,901,914 |  |

Table S2: Metadata summary for the dataset used in this study.

| **Species** | **Colony** | **Co-ordinates (°S; °W/E)** | **Oceanic frontal zone** | **Year** | **Number of full tracks** | |
| --- | --- | --- | --- | --- | --- | --- |
| SRHP | Beauchene Island | 52.95˚ S, 59.13˚ W | Southern Atlantic Ocean | 2011 | 32 |  |
| SRHP | Steeple Jason Island | 51.03˚ S, 61.21˚ W | Southern Atlantic Ocean | 2011 | 25 |  |
| SRHP | New Island | 51.71˚ S, 61.30˚ W | Southern Atlantic Ocean | 2012 | 11 |  |
| SRHP | Rugged Hill | 51.53˚ S, 57.86˚ W | Southern Atlantic Ocean | 2015 | 17 |  |
| SRHP | Bleaker Island | 52.26˚ S, 58.90˚ W | Southern Atlantic Ocean | 2014 | 16 |  |
| SRHP | Bleaker Island | 52.26˚ S, 58.90˚ W | Southern Atlantic Ocean | 2015 | 17 |  |
| SRHP | Pebble Island | 51.29˚ S, 59.64˚ W | Southern Atlantic Ocean | 2014 | 8 |  |
| SRHP | Pebble Island | 51.29˚ S, 59.64˚ W | Southern Atlantic Ocean | 2015 | 17 |  |
| SRHP | Cape Bougainvillea | 51.30˚ S, 58.47˚ W | Southern Atlantic Ocean | 2014 | 15 |  |
| SRHP | Cape Bougainvillea | 51.30˚ S, 58.47˚ W | Southern Atlantic Ocean | 2015 | 13 |  |
| SRHP | Diamond Cove | 51.54˚ S, 57.92˚ W | Southern Atlantic Ocean | 2014 | 15 |  |
| SRHP | Hummock Island | 51.61˚ S, 60.44˚ W | Southern Atlantic Ocean | 2020 | 14 |  |
| SRHP | Franklin Bay, Isla de los Estados | 54.81˚ S, 64.37˚ W | Southern Atlantic Ocean | 2020 | 15 |  |
| NRHP | Gough Island | 40.32˚ S, 9.93˚ W | Southern Atlantic Ocean | 2011 | 15 |  |
| NRHP | Gough Island | 40.32˚ S, 9.93˚ W | Southern Atlantic Ocean | 2012 | 27 |  |
| NRHP | Gough Island | 40.32˚ S, 9.93˚ W | Southern Atlantic Ocean | 2013 | 20 |  |
| NRHP | Nightingale Island | 37.42˚ S, 12.48˚ W | Southern Atlantic Ocean | 2017 | 29 |  |
| NRHP | Nightingale Island | 37.42˚ S, 12.48˚ W | Southern Atlantic Ocean | 2018 | 12 |  |
| NRHP | Amsterdam Island | 37.83˚ S, 77.55˚ E | Southern Indian Ocean | 2007 | 11 |  |
| ERHP | Marion Island | 46.90˚ S, 37.75˚ E | Southern Indian Ocean | 2013 | 17 |  |
| ERHP | Marion Island | 46.90˚ S, 37.75˚ E | Southern Indian Ocean | 2014 | 9 |  |
| ERHP | Crozet Island | 46.41˚ S, 51.98˚ E | Southern Indian Ocean | 2007 | 11 |  |
| ERHP | Kerguelen Island | 49.33˚ S, 70.33˚ E | Southern Indian Ocean | 2007 | 14 |  |
| ERHP | Campbell Island | 52.54˚ S, 169.14˚ E | Southern Pacific Ocean | 2013 | 30 |  |
| MRP | Bird Island | 54.00˚ S, 38.04˚ W | Southern Atlantic Ocean | 2011 | 31 |  |
| MRP | Marion Island | 46.90˚ S, 37.75˚ E | Southern Indian Ocean | 2014 | 17 |  |
| MRP | Kerguelen Island | 49.33˚ S, 70.33˚ E | Southern Indian Ocean | 2006 | 10 |  |
| MRP | Kerguelen Island | 49.33˚ S, 70.33˚ E | Southern Indian Ocean | 2007 | 6 |  |
| MRP | Crozet Island | 46.41˚ S, 51.98˚ E | Southern Indian Ocean | 2007 | 11 |  |

SHRP = Southern rockhopper penguin, NRHP = Northern rockhopper penguin, ERHP = Eastern rockhopper penguin, MRP = Macaroni penguin

Table S3: Tracking data used in this study, the data owners and the approval ethics permit/committees

| Species | Colony | Year of data collection | Data owners | Ethics approval | Publications and Data availability link |
| --- | --- | --- | --- | --- | --- |
| SRHP | Beauchene Island | 2011 | Norman Ratcliffe | Permission for the fieldwork was granted by the Falklands and South Georgia governments and the Wildlife Conservation Society. Animal welfare was overseen by the British Antarctic Survey Animal Ethics Committee. | Ratcliffe et al., 2014  BirdLife International Seabird Tracking Database  <http://seabirdtracking.org/mapper/?dataset_id=763> |
| SRHP | Steeple Jason | 2011 | Norman Ratcliffe | Permission for the fieldwork was granted by the Falklands and South Georgia governments and the Wildlife Conservation Society. Animal welfare was overseen by the British Antarctic Survey Animal Ethics Committee. | Ratcliffe et al., 2014  BirdLife International Seabird Tracking Database  <http://seabirdtracking.org/mapper/?dataset_id=778> |
| SRHP | New Island | 2012 | Charles-André Bost,  Jean-Baptiste Thiebot, Maud Poisbleau, Laurent Demongin | Research licence granted by the Environmental Planning Department of the Falkland Islands Government (research licence numbers: R06/2009 and R14.2011)”, and “The study was performed according to Belgian and Flemish law and was approved by the ethical committee on animal experimentation (ECD, ID number: 2011/44). | Thiebot et al. 2015  BirdLife International Seabird Tracking Database  <http://seabirdtracking.org/mapper/?dataset_id=947> |
| SRHP | Rugged Hill | 2015 | Megan Tierney, Alastair Baylis | Falkland Islands Government | BirdLife International Seabird Tracking Database  <http://seabirdtracking.org/mapper/?dataset_id=1903> |
| SRHP | Bleaker Island | 2014 | Megan Tierney, Alastair Baylis | Falkland Islands Government | BirdLife International Seabird Tracking Database  <http://seabirdtracking.org/mapper/?dataset_id=1874> |
| SRHP | Bleaker Island | 2015 | Megan Tierney, Alastair Baylis | Falkland Islands Government | BirdLife International Seabird Tracking Database  <http://seabirdtracking.org/mapper/?dataset_id=1897> |
| SRHP | Pebble Island | 2014 | Megan Tierney, Alastair Baylis | Falkland Islands Government | BirdLife International Seabird Tracking Database  <http://seabirdtracking.org/mapper/?dataset_id=1901> |
| SRHP | Pebble Island | 2015 | Megan Tierney, Alastair Baylis | Falkland Islands Government | BirdLife International Seabird Tracking Database  <http://seabirdtracking.org/mapper/?dataset_id=1902> |
| SRHP | Cape Bougainvillea | 2014 | Megan Tierney, Alastair Baylis | Falkland Islands Government | BirdLife International Seabird Tracking Database  <http://seabirdtracking.org/mapper/?dataset_id=1898> |
| SRHP | Cape Bougainvillea | 2015 | Megan Tierney, Alastair Baylis | Falkland Islands Government | BirdLife International Seabird Tracking Database  <http://seabirdtracking.org/mapper/?dataset_id=1899> |
| SRHP | Diamond Cove | 2014 | Megan Tierney, Alastair Baylis | Falkland Islands Government | BirdLife International Seabird Tracking Database  <http://seabirdtracking.org/mapper/?dataset_id=1900> |
| SRHP | Hummock Island | 2020 | Klemens Pütz | Falkland Islands Government and University of Tasmania Animal Ethics Committee | BirdLife International Seabird Tracking Database  <http://seabirdtracking.org/mapper/?dataset_id=1877> |
| SRHP | Franklin Bay, Isla de los Estados | 2020 | Andrea Raya Rey, Klemens Pütz | Falkland Islands Government and University of Tasmania Animal Ethics Committee | BirdLife International Seabird Tracking Database <http://seabirdtracking.org/mapper/?dataset_id=1876> |
| NRHP | Gough | 2011 | Richard Cuthbert,  Jacob Gonzalez-Solis,  Peter G. Ryan | Faculty of Science Animal Ethics Committee (SFAEC), University of Cape Town & Tristan da Cunha government | BirdLife International Seabird Tracking Database  <http://seabirdtracking.org/mapper/?dataset_id=1887> |
| NRHP | Gough | 2012 | Richard Cuthbert,  Jacob Gonzalez-Solis,  Peter G. Ryan | Faculty of Science Animal Ethics Committee (SFAEC), University of Cape Town & Tristan da Cunha government | BirdLife International Seabird Tracking Database  <http://seabirdtracking.org/mapper/?dataset_id=1886> |
| NRHP | Gough | 2013 | Antje Steinfurth,  Richard Cuthbert,  Jacob Gonzalez-Solis, Peter G. Ryan | Faculty of Science Animal Ethics Committee (SFAEC), University of Cape Town & Tristan da Cunha government | BirdLife International Seabird Tracking Database  <http://seabirdtracking.org/mapper/?dataset_id=1883> |
| NRHP | Nightingale | 2017 | Antje Steinfurth, Alex Bond, Norman Ratcliffe, Trevor Glass | British Trust for Ornithology’s Special Marks Technical Panel  & Tristan da Cunha Government | BirdLife International Seabird Tracking Database  <http://seabirdtracking.org/mapper/?dataset_id=1884> |
| NRHP | Nightingale | 2018 | Antje Steinfurth, Alex Bond, Norman Ratcliffe, Trevor Glass | British Trust for Ornithology’s Special Marks Technical Panel, Tristan da Cunha Government & BAS Animal Welfare and Ethical Review Body | BirdLife International Seabird Tracking Database  <http://seabirdtracking.org/mapper/?dataset_id=1881> |
| NRHP | Amsterdam | 2007 | Charles-André Bost,  Jean-Baptiste Thiebot | The Ethics Committee of the Institut Polaire Français Paul-Emile Victor (IPEV) approved the field procedure. | Thiebot et al., 2013  CEBc-CNRS data archived at Birdlife Repository. Contact Charles-André Bost [charly.bost@cebc.cnrs.fr](mailto:charly.bost@cebc.cnrs.fr) for access. |
| ERHP | Marion Island | 2013 | T. Otto Whitehead, Peter G. Ryan,  Fitzpatrick Institute of Ornithology, University of Cape Town | All procedures performed in this study were in accordance with the ethical standards of the Faculty of Science Animal Ethics Committee (SFAEC), University of Cape Town (2013/V5/ NEW). | Whitehead et al. 2016  BirdLife International Seabird Tracking Database  <http://seabirdtracking.org/mapper/?dataset_id=1878> |
| ERHP | Marion Island | 2014 | T. Otto Whitehead, Peter G. Ryan,  Fitzpatrick Institute of Ornithology, University of Cape Town | All procedures performed in this study were in accordance with the ethical standards of the Faculty of Science Animal Ethics Committee (SFAEC), University of Cape Town (2013/V5/ NEW). | Whitehead et al. 2016  BirdLife International Seabird Tracking Database  <http://seabirdtracking.org/mapper/?dataset_id=1879> |
| ERHP | Crozet | 2007 | Charles-André Bost,  Jean-Baptiste Thiebot | The Ethics Committee of the Institut Polaire Français Paul-Emile Victor (IPEV) approved the field procedure. | Thiebot et al., 2013  CEBc-CNRS data archived at Birdlife Repository. Contact Charles-André Bost [charly.bost@cebc.cnrs.fr](mailto:charly.bost@cebc.cnrs.fr) for access. |
| ERHP | Kerguelen | 2007 | Charles-André Bost,  Jean-Baptiste Thiebot | The Ethics Committee of the Institut Polaire Français Paul-Emile Victor (IPEV) approved the field procedure. | Thiebot et al., 2013  CEBc-CNRS data archived at Birdlife Repository. Contact Charles-André Bost [charly.bost@cebc.cnrs.fr](mailto:charly.bost@cebc.cnrs.fr) for access. |
| ERHP | Campbell | 2013 | Norman Ratcliffe, David Thompson | New Zealand Department of Conservation and the National Institute for Water and Atmospheric research | BirdLife International Seabird Tracking Database  <http://seabirdtracking.org/mapper/?dataset_id=1875> |
| MRP | Bird Island | 2011 | Norman Ratcliffe | Permission for the fieldwork was granted by the Falklands and South Georgia governments and the Wildlife Conservation Society. Animal welfare was overseen by the British Antarctic Survey Animal Ethics Committee. | Ratcliffe et al., 2014  BirdLife International Seabird Tracking Database  <http://seabirdtracking.org/mapper/?dataset_id=742> |
| MRP | Marion Island | 2014 | T. Otto Whitehead, Peter G. Ryan,  Fitzpatrick Institute of Ornithology, University of Cape Town | All procedures performed in this study were in accordance with the ethical standards of the Faculty of Science Animal Ethics Committee (SFAEC), University of Cape Town (2013/V5/ NEW). | Whitehead et al. 2016  BirdLife International Seabird Tracking Database  <http://seabirdtracking.org/mapper/?dataset_id=1880> |
| MRP | Kerguelen | 2006 | Charles-André Bost,  Jean-Baptiste Thiebot | The Ethics Committee of the Institut Polaire Français Paul-Emile Victor (IPEV) approved the field procedure. | Thiebot et al., 2013  BirdLife International Seabird Tracking Database  <http://seabirdtracking.org/mapper/?dataset_id=740> |
| MRP | Kerguelen | 2007 | Charles-André Bost,  Jean-Baptiste Thiebot | The Ethics Committee of the Institut Polaire Français Paul-Emile Victor (IPEV) approved the field procedure. | Thiebot et al., 2013  CEBc-CNRS data archived at Birdlife Repository. Contact Charles-André Bost [charly.bost@cebc.cnrs.fr](mailto:charly.bost@cebc.cnrs.fr) for access. |
| MRP | Crozet | 2007 | Charles-André Bost,  Jean-Baptiste Thiebot | The Ethics Committee of the Institut Polaire Français Paul-Emile Victor (IPEV) approved the field procedure. | Thiebot et al., 2013  CEBc-CNRS data archived at Birdlife Repository. Contact Charles-André Bost [charly.bost@cebc.cnrs.fr](mailto:charly.bost@cebc.cnrs.fr) for access. |

SHRP = Southern rockhopper penguin, NRHP = Northern rockhopper penguin, ERHP = Eastern rockhopper penguin, MRP = Macaroni penguin


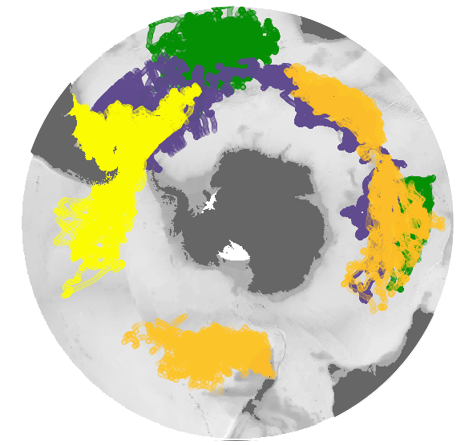


Fig. S1. GLS tracking data used in this study to model *Eudyptes* penguin habitat preference during the pre-moult exodus for: eastern rockhopper (E. filholi; orange), macaroni penguins (E. chrysolophus; purple), northern rockhopper (E. moseleyi; green), and southern rockhopper (E. chrysocome; yellow) penguins.

Table S4: Environmental covariates used as predictors to model *Eudyptes* penguin habitat preference during the pre-moult migrations.

| Environmental covariate | Variable full name | Spatial Resolution | Temporal Resolution | Unit | Extraction | Source |
| --- | --- | --- | --- | --- | --- | --- |
| SSH | Sea surface height | 0.083 ˚ x 0.083 ˚ | daily-mean | m | timestep | Global_Reanalysis_Phy_001_030, Copernicus |
| SSHa | Sea surface height anomaly | 0.083 ˚ x 0.083 ˚ | daily-mean | m | timestep | Global_Reanalysis_Phy _001_030, Copernicus |
| Bathy | Depth | 0.083 ˚ x 0.083 ˚ | Static | m | timestep | Global_Reanalysis_Phy _001_030, Copernicus |
| Bathyg | Gradient depth | 0.083 ˚ x 0.083 ˚ | Static | ˚ | timestep | Global_Reanalysis_Phy _001_030, Copernicus |
| SST | Sea surface temperature | 0.083 ˚ x 0.083 ˚ | daily-mean | ˚C | timestep | Global_Reanalysis_Phy _001_030, Copernicus |
| Chla | Chlorophyll *a* concentration (surface) | 0.25 ˚ x 0.25 ˚ | daily-mean | mg.m^-3^ | timestep | Global_Reanalysis_Phy _001_029, Copernicus |
| EKE | Eddy kinetic energy | 0.083 ˚ x 0.083 ˚ | daily-mean | cm^2^.s^-2^ | timestep | Global_Reanalysis_Phy _001_030, Copernicus |
| MLD | Mixed layer depth | 0.083 ˚ x 0.083 ˚ | daily-mean | m | timestep | Global_Reanalysis_Phy _001_030, Copernicus |
| Ice | Ice with concentration >80% | 0.25 ˚ x 0.25 ˚ | weekly-mean | % | climatology | Global_Reanalysis_Phy _001_029, Copernicus |

Table S5. Model metrics for boosted regression tree models that model habitat preference for a) eastern rockhopper penguin, b) macaroni penguin, c) northern rockhopper penguin and d) southern rockhopper penguin across the Southern Ocean. For each colony we provide the cross-validation correlation (cv Mean) and the standard error (cv S.E) as well as the proportion of predictions that are True Negatives (i.e. correctly assigned as bird not-present), False Negatives (incorrectly assigned as bird not-present), False Positives (incorrectly assigned as bird present) and True Positive (correctly assigned as bird present).

| 1. **eastern rockhopper** | | | | | | |
| --- | --- | --- | --- | --- | --- | --- |
|  | **Model metrics** | | **External Cross-validation** | | | |
| **Colony** | Cv  Mean | Cv  S.E. | True  Negative | False  Negative | False  Positive | True  Positive |
| **Crozet** | 0.57 | 0.02 | 0.99 | 0.01 | 0.5 | 0.50 |
| **Campbell** | 0.45 | 0.01 | 0.60 | 0.40 | 0.25 | 0.75 |
| **Kerguelen** | 0.51 | 0.02 | 0.99 | 0.01 | 0.45 | 0.55 |
| **Marion** | 0.57 | 0.02 | 0.90 | 0.10 | 0.46 | 0.54 |
| **Mean** | 0.53 | 0.01 | 0.87 | 0.13 | 0.41 | 0.59 |
| **S.D.** | 0.05 | 0.01 | 0.18 | 0.18 | 0.11 | 0.11 |

| 1. **macaroni penguin** | | | | | | |
| --- | --- | --- | --- | --- | --- | --- |
|  | **Model metrics** | | **External Cross-validation** | | | |
| **Colony** | Cv  Mean | Cv  S.E. | True  Negative | False  Negative | False Positive | True  Positive |
| **Bird** | 0.39 | 0.02 | 0.90 | 0.10 | 0.45 | 0.55 |
| **Crozet** | 0.75 | 0.01 | 0.85 | 0.15 | 0.30 | 0.70 |
| **Kerguelen** | 0.75 | 0.02 | 0.84 | 0.16 | 0.27 | 0.73 |
| **Marion** | 0.71 | 0.01 | 0.82 | 0.18 | 0.32 | 0.68 |
| **Mean** | 0.65 | 0.02 | 0.85 | 0.15 | 0.34 | 0.67 |
| **S.D.** | 0.17 | 0.01 | 0.03 | 0.03 | 0.08 | 0.08 |

| 1. **northern rockhopper** | | | | | | |
| --- | --- | --- | --- | --- | --- | --- |
|  | **Model metrics** | | **External Cross-validation** | | | |
| **Colony** | Cv  Mean | Cv  S.E. | True  Negative | False  Negative | False Positive | True  Positive |
| **Amsterdam** | 0.44 | 0.01 | 0.53 | 0.47 | 0.55 | 0.45 |
| **Gough** | 0.57 | 0.02 | 0.95 | 0.05 | 0.39 | 0.61 |
| **Nightingale** | 0.56 | 0.01 | 0.98 | 0.02 | 0.32 | 0.68 |
| **Mean** | 0.52 | 0.01 | 0.82 | 0.18 | 0.42 | 0.58 |
| **S.D.** | 0.06 | 0.01 | 0.25 | 0.25 | 0.12 | 0.12 |

| 1. **southern rockhopper** | | | | | | |
| --- | --- | --- | --- | --- | --- | --- |
|  | **Model metrics** | | **External Cross-validation** | | | |
| **Colony** | Cv  Mean | Cv  S.E. | True  Negative | False  Negative | False Positive | True  Positive |
| **Falkland Islands (Malvinas)** | 0.55 | 0.02 | 0.90 | 0.10 | 0.48 | 0.52 |
| **Isla de los Estados** | 0.55 | 0.02 | 0.94 | 0.06 | 0.42 | 0.58 |
| **Mean** | 0.55 | 0.02 | 0.92 | 0.08 | 0.45 | 0.55 |
| **S.D.** | 0 | 0 | 0.02 | 0.02 | 0.03 | 0.03 |

Table S6.1: Variables in order of importance with percentage contribution to the habitat preference model for eastern rockhopper penguin tracking data used in this study.

| Bathy | 34.8 % |
| --- | --- |
| SSH | 17.1 % |
| SST | 11.8 % |
| MLD | 10.3 % |
| Chla | 8.0 % |
| Bathyg | 7.5 % |
| EKE | 5.3 % |
| SSHa | 5.0 % |
| Ice | 0.0 % |

Table S6.2: Variables in order of importance with percentage contribution to the habitat preference model for macaroni penguin tracking data used in this study.

| SST | 80.4 % |
| --- | --- |
| Bathy | 3.5 % |
| EKE | 3.2 % |
| MLD | 3.1 % |
| Bathyg | 2.7 % |
| SSH | 2.7 % |
| Chla | 2.4 % |
| SSHa | 2.0 % |
| Ice | 0.0 % |

Table S6.3: Variables in order of importance with percentage contribution to the habitat preference model for northern rockhopper penguin tracking data used in this study.

| SST | 23.6 % |
| --- | --- |
| SSH | 21.9 % |
| SSHa | 14.0 % |
| Bathy | 10.9 % |
| EKE | 9.4 % |
| Chla | 8.5 % |
| MLD | 6.4 % |
| Bathyg | 5.3 % |
| Ice | 0.0 % |

Table S6.4: Variables in order of importance with percentage contribution to the habitat preference model for southern rockhopper penguin tracking data used in this study.

| SSH | 25.6 % |
| --- | --- |
| MLD | 21.8 % |
| SST | 14.3 % |
| Chla | 14.2 % |
| EKE | 12.8 % |
| SSHa | 5.9 % |
| Ice | 5.4 % |
| Bathy | 0.0 % |
| Bathyg | 0.0 % |

eastern rockhopper penguin


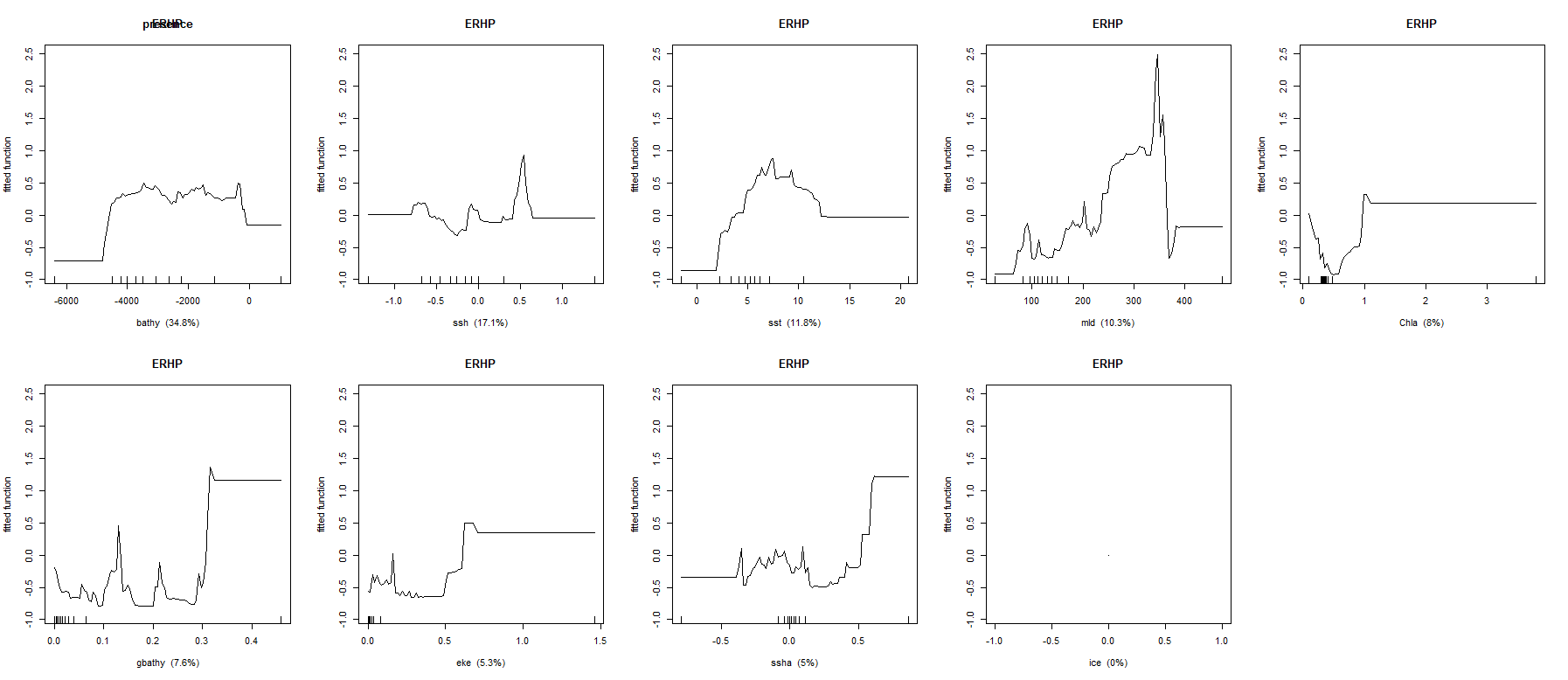


macaroni penguin


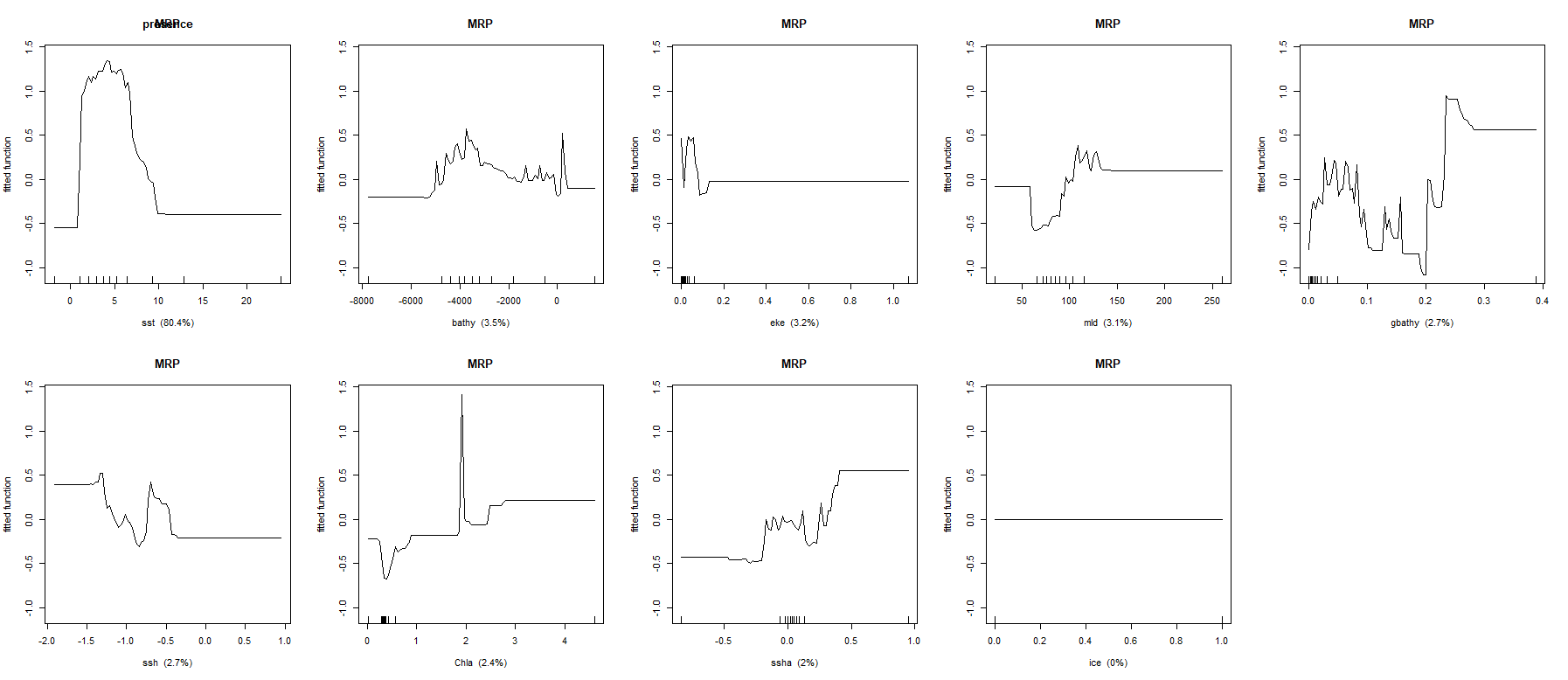


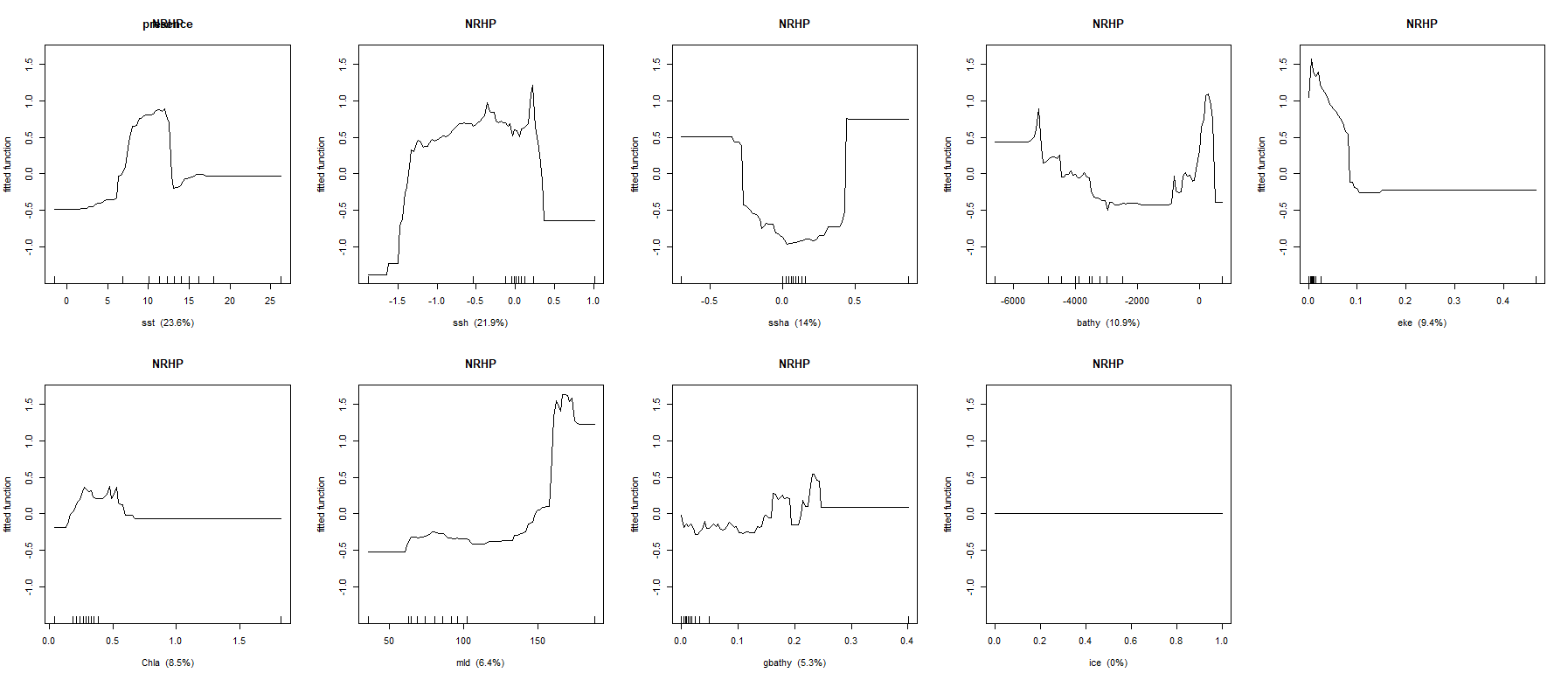


northern rockhopper penguin


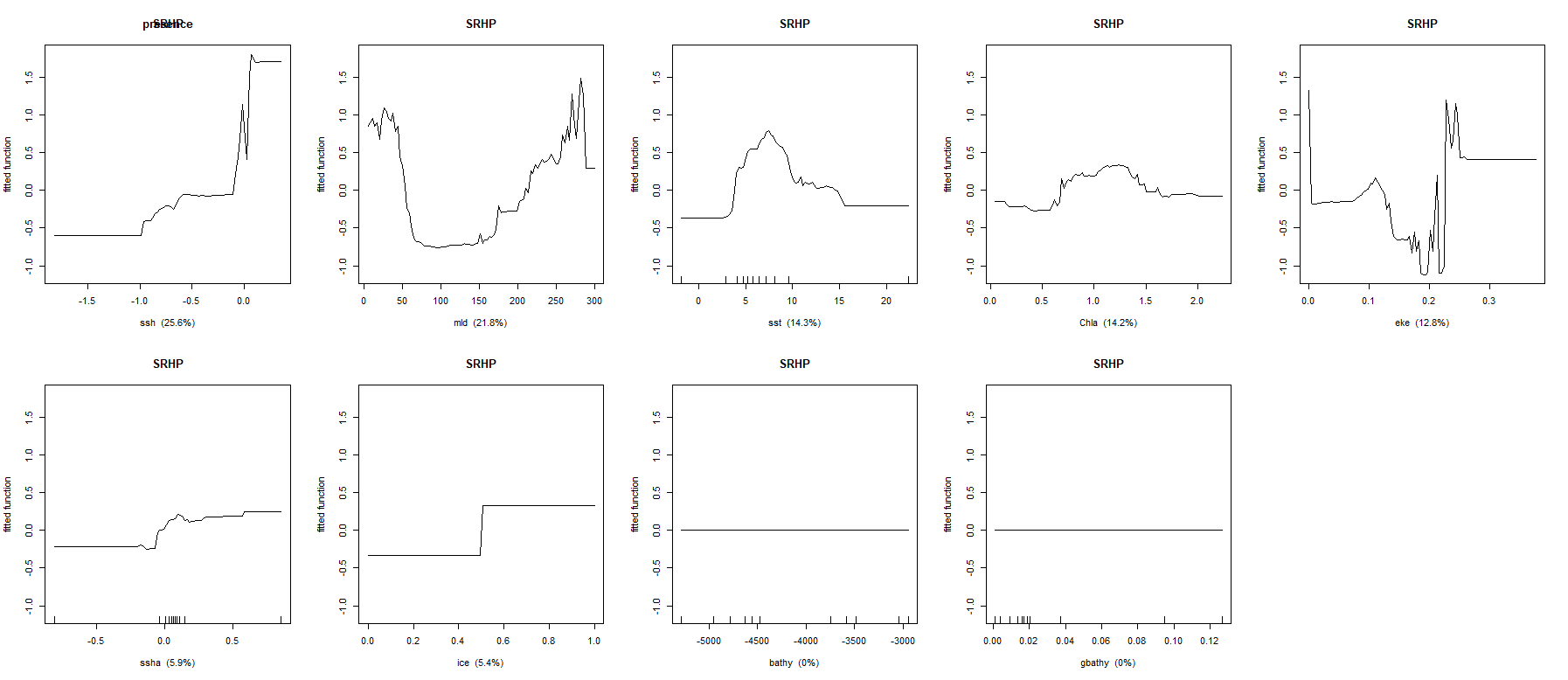


southern rockhopper penguin

Figure S2: Response curves for eastern rockhopper (E. filholi), macaroni (E. chrysolophus)/royal (E. schlegeli) penguins, northern rockhopper (E. moseleyi), and southern rockhopper (E. chrysocome) penguin for full suite of predictor variables in the current habitat preference models.


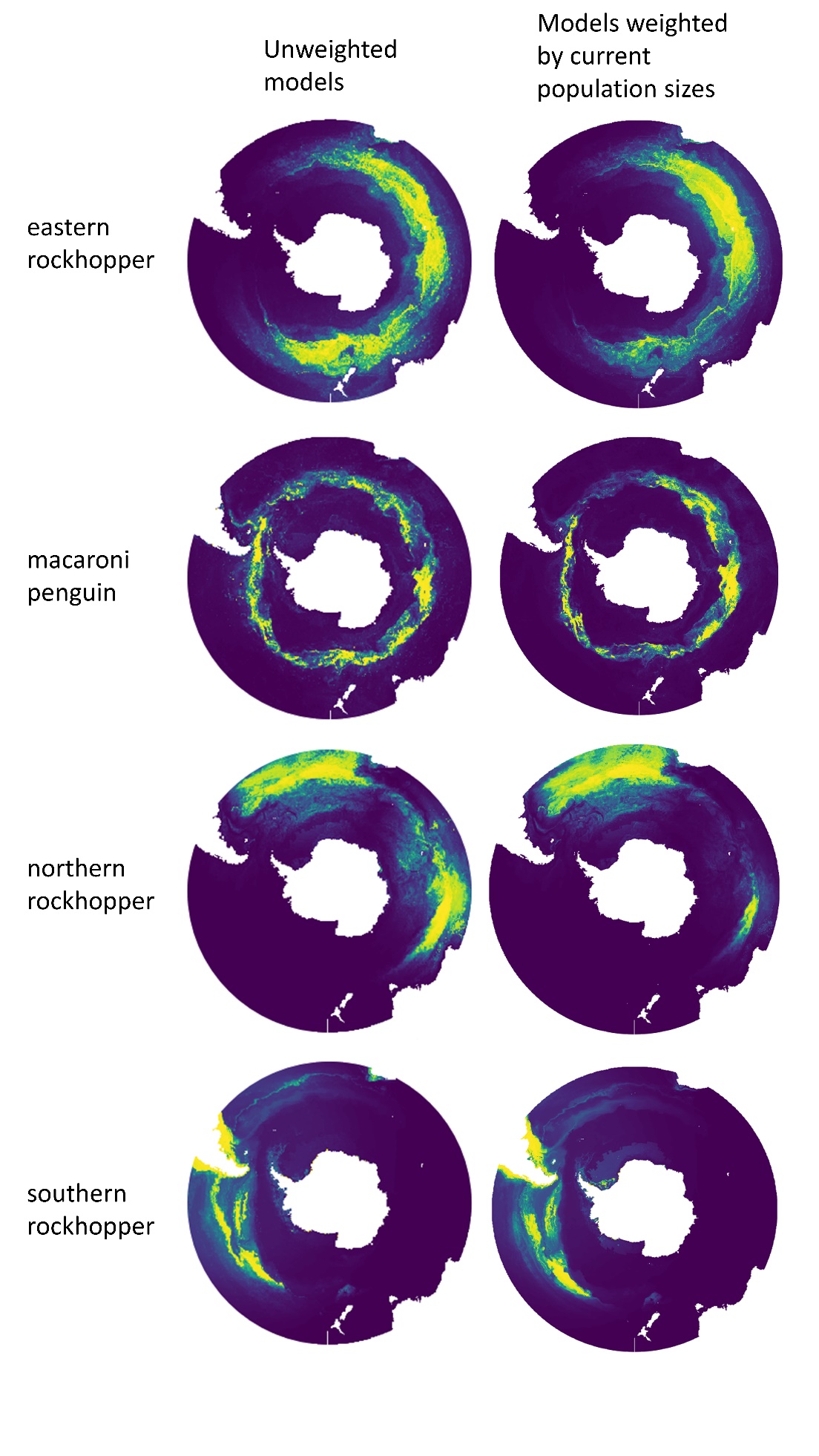


Figure S3: Comparison between habitat preference models for eastern rockhopper (*E. filholi*), macaroni (*E. chrysolophus*)/royal (*E. schlegeli*) penguins, northern rockhopper (*E. moseleyi*), and southern rockhopper (*E. chrysocome*) penguins for unweighted models and models weighted by population size (as in shown in Table 1 in this same document).


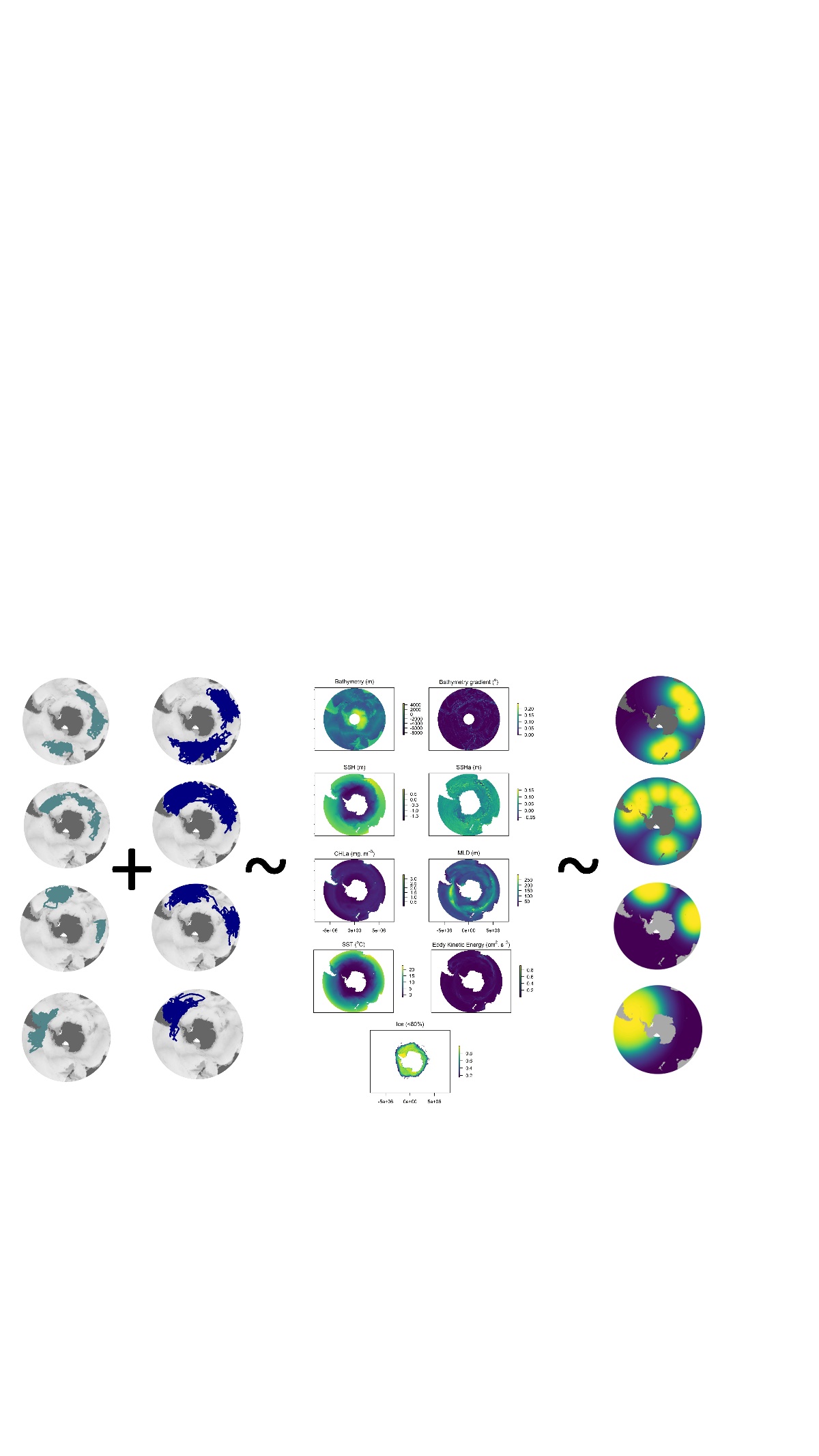


D

C

B

A

iv

iii

ii

i


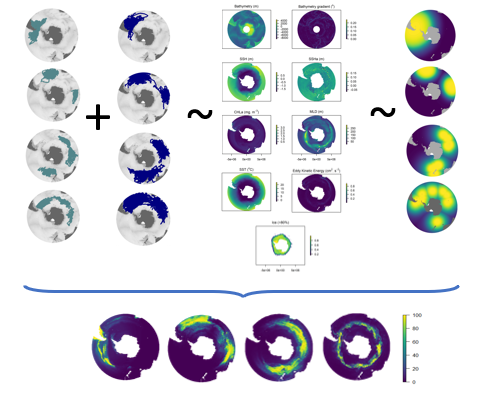


E

Fig. S4. Schematic presentation of current habitat preference modelling methodology. i) Eastern rockhoppers (*E. filholi*), ii) macaroni penguins (*E. chrysolophus*), iii) northern rockhoppers (*E. moseleyi*) and iv) southern rockhoppers (*E. chrysocome*) penguins were tracked using light geolocation devices. Observed tracks (A) and simulated/pseudo-absence tracks (B) were modelled with a suite of 9 environmental variables (C). Habitat selection models were constrained by distances that the species could feasibly go from their colonies (D). The final outputs were scaled from 0 – 100 to be comparable across taxa (E).

Table S7. List of climate change models (CMIP5 representations) and variables used to predict future climate redistributions (source of figure: Hindell et al., 2020 Supplementary material page 20). The variables used were sea ice concentration (sic), sea surface temperature (tos), sea surface salinity (sos), sea surface height (zos), the spatial gradient of sea surface height (zosgrad), near-surface current speed (curr), near-surface wind speed (wind), and surface downward heat flux (hfds). O (green) blocks denote where data were available and X (red) blocks represent where no data were available.


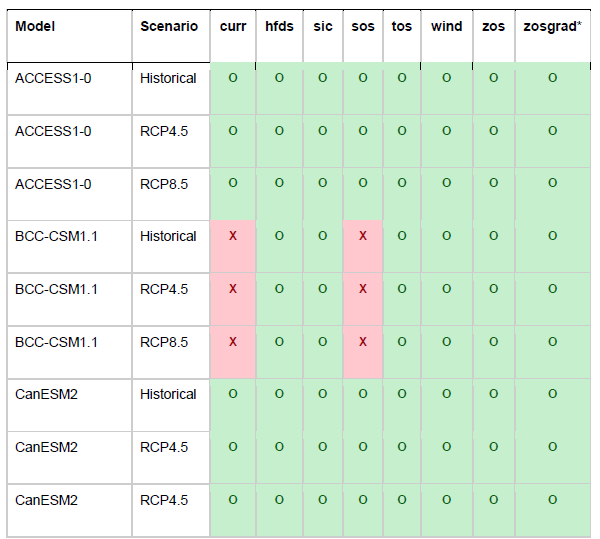


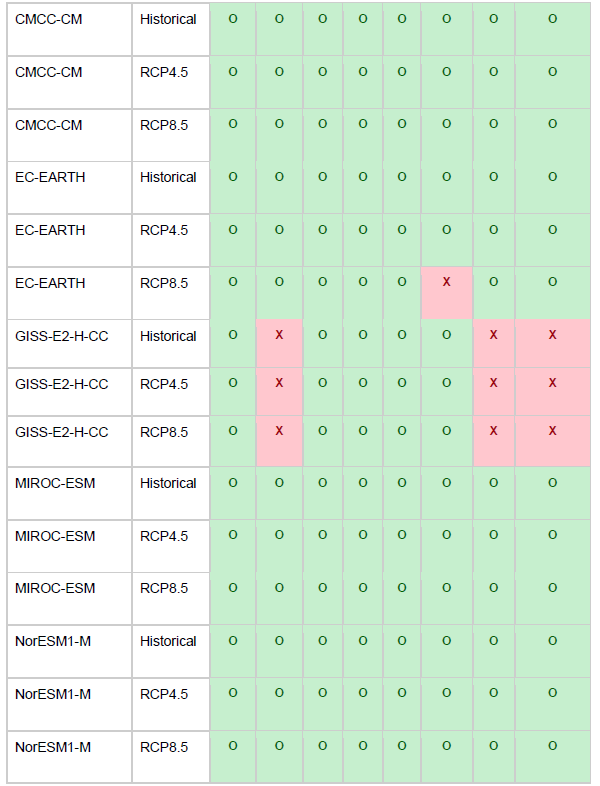


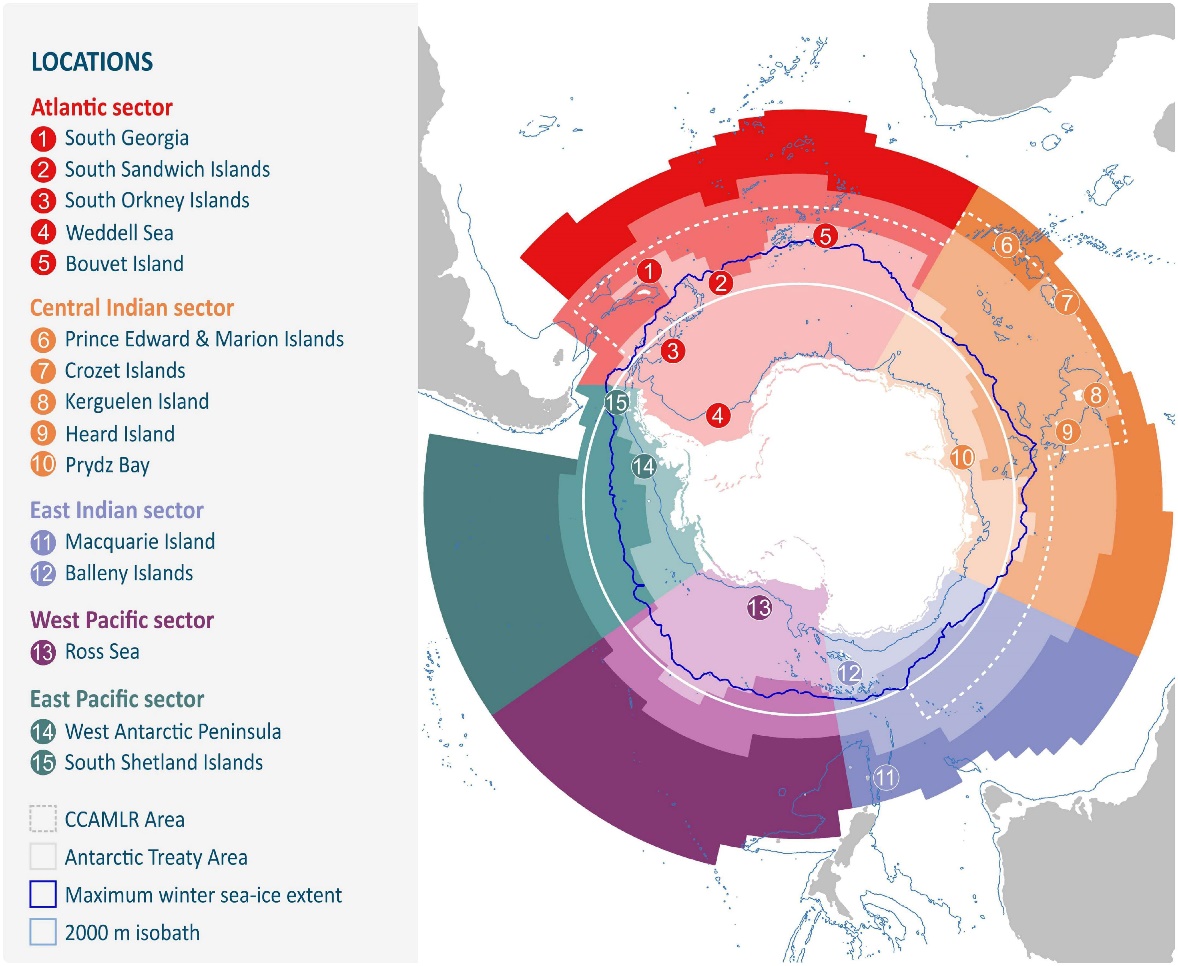


Fig. S5. Regions of the Southern Ocean defined by the Marine Ecosystem Assessment for the Southern Ocean: Atlantic, Central Indian, East Indian, West Pacific and East Pacific (Source of figure: McCormack et al., 2021).

Code used to calculate percentage habitat change by Marine Ecosystem Assessment for the Southern Ocean (MEASO) defined region.

# # # # # # # # # # # # # # # # # #

# Description: Calculating percentage habitat change by MEASO defined region

# Author: David B. Green

# Date: 20-02-2022

# # # # # # # # # # # # # # # # # #

library(raster)

library(measoshapes)

library(sf)

library(tidyverse)

library(fs)

# Get RDS files

rf <- dir_ls("Results/", glob = "*multimodel*pca*measo*.rds")

rdat_aes <- lapply(rf, function(x){

sp = str_split(x, pattern = "_")[[1]][1]

sp = str_replace(sp, pattern = "Results/", replacement = "")

scen = str_split(x, pattern = "_")[[1]][3]

rdat = readr::read_rds(x)[["projected_aes"]] %>%

mutate(species = sp,

scenario = scen)

}) %>%

bind_rows() %>%

filter(core_area > 30000)

rdat_all <- rdat_aes %>%

group_by(species, model, scenario) %>%

summarise(area = sum(area),

core_area = sum(core_area),

area_prop = (area-core_area)/core_area,

sector = "All")

rdat_aes <- bind_rows(rdat_aes, rdat_all)

rdat_aes_sum <- rdat_aes %>%

group_by(species,scenario,sector) %>%

summarise(sdarea = sd(area),

area = mean(area),

sdcore_area = sd(core_area),

core_area = mean(core_area),

sdarea_prop = sd(area_prop),

area_prop = mean(area_prop)

)

readr::write_csv(rdat_aes_sum, file = paste("summary_table_for_eudyptes_perc_habitat_change_cmip5_",Sys.Date(),".csv"))

g1 <- ggplot(rdat_aes,

mapping = aes(x = (as.factor(sector)),

y = area_prop*100,

# group = as.factor(species),

fill = factor(scenario),

colour = factor(scenario)

# fill = factor(species),

# colour = factor(species)

)

) +

geom_boxplot(

# mapping = aes(x = (factor(sector)),

# y = area_prop*100

# #fill = factor(sector)

# ),

# fill = "grey70",

# colour = "grey60",

notch = F,

notchwidth = 0.05,

# width = 0.2,

outlier.colour = NA

) +

geom_jitter(

# mapping = aes(colour = scenario#,

# #position = scenario

# ),

position = position_jitterdodge(

),

colour = "grey60"

# width = 0.1

) +

# stat_summary(aes(colour = factor(scenario)),

# # position = position_jitterdodge(),

# fun.y=mean,

# geom="point",

# shape=20,

# size=6,

# color="red",

# fill="red"

#

# ) +

geom_hline(yintercept = 0,

size = 1,

# lty = "dotted",

colour = "grey60") +

labs(y = "Projected change in area (%)",

x = "Ocean sector",

fill = "Scenario",

colour = "Scenario"

) +

scale_x_discrete(

labels = (c("West Pacific", "East Pacific", "East Indian", "Central Indian", "Atlantic", "All")),

# limits = rev(levels(factor(sector)))

limits = rev

) +

scale_y_continuous(

limits = c(-100,100)

) +

coord_flip() +

facet_wrap(~species, ncol=2) +

theme(

panel.background = element_blank(),

panel.border = element_rect(fill = NA),

text = element_text(

size = 18

)

)

ggsave("Relatively a difference in areas of penguin places that are hip and happening.png",

g1, device = "png", width = 30, height = 26, units = "cm", dpi = 300)

### Not as a proportion

rdat_diff <- rdat_aes %>%

mutate(area_diff = area - core_area)

g2 <- ggplot(rdat_diff,

mapping = aes(x = (as.factor(sector)),

y = area_diff,

# group = as.factor(species),

fill = factor(scenario),

colour = factor(scenario)

# fill = factor(species),

# colour = factor(species)

)

) +

geom_boxplot(

# mapping = aes(x = (factor(sector)),

# y = area_prop*100

# #fill = factor(sector)

# ),

# fill = "grey70",

# colour = "grey60",

notch = F,

notchwidth = 0.05,

# width = 0.2,

outlier.colour = NA

) +

geom_jitter(

# mapping = aes(colour = scenario#,

# #position = scenario

# ),

position = position_jitterdodge(

),

colour = "grey60"

# width = 0.1

) +

# stat_summary(aes(colour = factor(scenario)),

# # position = position_jitterdodge(),

# fun.y=mean,

# geom="point",

# shape=20,

# size=6,

# color="red",

# fill="red"

#

# ) +

geom_hline(yintercept = 0,

size = 1,

# lty = "dotted",

colour = "grey60") +

labs(y = expr(paste("Projected change in area (km"^"2",")")),

x = "Ocean sector",

fill = "Scenario",

colour = "Scenario"

) +

scale_x_discrete(

labels = (c("West Pacific", "East Pacific", "East Indian", "Central Indian", "Atlantic", "All")),

# limits = rev(levels(factor(sector)))

limits = rev

) +

scale_y_continuous(

# limits = c(-100,100)

) +

coord_flip() +

facet_wrap(~species, ncol=2) +

theme(

panel.background = element_blank(),

panel.border = element_rect(fill = NA),

text = element_text(

size = 18

)

)

ggsave("absolutely a difference in areas of penguin places that are hip and happening.png",g2,

device = "png", width = 30, height = 26, units = "cm", dpi = 300)


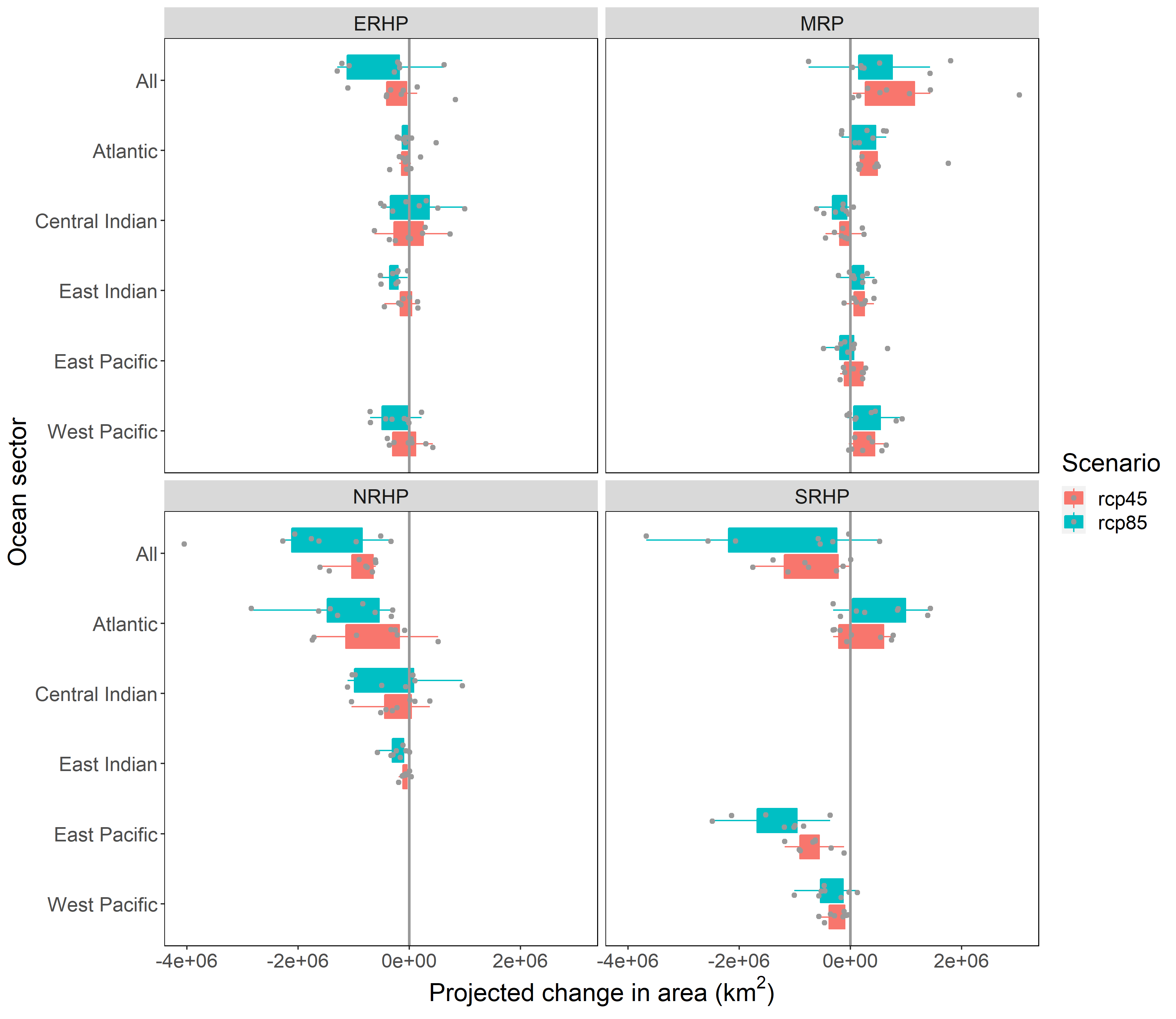


Fig. S6. Projected change in habitat area per region (using the Marine Ecosystem Assessment for the Southern Ocean defined regions) for the eight different climate models (ACCESS1.0, BCC-CSM1.1, CanESM2, CMCC-CM, EC-EARTH, GISS-E2-H-CC, MIROC-ESM, and NorESM-M: grey points) for eastern rockhoppers (ERHP), macaroni penguin (MRP), northern rockhoppers (NRHP) and southern rockhopper penguins (SRHP) under Representative Concentration Pathways RCP4.5 (red) and RCP8.5 (blue) scenarios. The boxes indicate the 25^th^ – 75^th^ percentiles.

**References**

Barbraud, C. et al. 2020. Population trends of penguins in the French Southern Territories. - Polar Biol. 43: 835–850.

Baylis, A. M. M. et al. 2013. Increasing trend in the number of Southern Rockhopper Penguins (Eudyptes c. chrysocome) breeding at the Falkland Islands. - Polar Biol. 36: 1007–1018.

Birdlife 2018. State of the world’s birds: taking the pulse of the planet.

BirdLife International. 2020. 2020. No Title. - Eudyptes moseleyi. IUCN Red List Threat. Species 2020 e.T22734408A184698049. https//dx.doi.org/10.2305/IUCN.UK.2020-3.RLTS.T22734408A184698049.en. Accessed 11 May 2022.

Cooper, J. et al. 1990. Diets and Dietary Segregation of Crested Penguins (Eudyptes). - In: Penguin Biology. Academic Press, pp. 131–156.

Crawford, R. J. M. et al. 2009. Recent trends in numbers of four species of penguins at the Prince Edward Islands. - African J. Mar. Sci. 31: 419–426.

Crossin, G. T. et al. 2013. Macaroni Penguin (Eudyptes chrysolophus) and Royal Penguin (Eudyptes schlegeli). in press.

Cuthbert, R. et al. 2009. Population trends and conservation status of the Northern Rockhopper Penguin Eudyptes moseleyi at Tristan da Cunha and Gough Island. - Bird Conserv. Int. 19: 109–120.

Gandini, P. et al. 2017. Population trends of the Southern Rockhopper Penguin (Eudyptes chrysocome chrysocome) at the northern limit of its breeding range: Isla Pingüino, Santa Cruz, Argentina. - Polar Biol. 40: 1023–1028.

Hindell, M. A. et al. 2020. Tracking of marine predators to protect Southern Ocean ecosystems. - Nature 580: 87–92.

Hiscock, J. A. and Chilvers, B. L. 2014. Declining eastern rockhopper (Eudyptes flholi) and erect-crested (E. sclateri) penguins on the Antipodes Islands, New Zealand. - N. Z. J. Ecol. 38: 124–131.

Horswill, C. 2015. The relative importance of opposing drivers in determining population change in macaroni penguins Eudyptes chrysolophus. Ph.D. Thes

Kirkwood, R. et al. 2007. Estimates of southern rockhopper and macaroni penguin numbers at the Ildefonso and Diego Ramírez Archipelagos, Chile, using quadrat and distance-sampling techniques. - Waterbirds 30: 259–267.

McCormack, S. A. et al. 2021. Southern Ocean food web modelling: progress, prognoses, and future priorities for research and policy makers. - Front. Ecol. Evol.: 626.

Morrison, K. W. et al. 2015. Population dynamics of Eastern Rockhopper Penguins on Campbell Island in relation to sea surface temperature 1942–2012: current warming hiatus pauses a long-term decline. - Polar Biol. 38: 163–177.

Niemandt, C. et al. 2015. Chinstrap and macaroni penguin diet and demography at Nyroysa, Bouvetoya. - Antarct. Sci. 28: 91–100.

Oehler, D. A. et al. 2008. Status of Crested Penguin (Eudyptes spp.) populations on three islands in Southern Chile. - Wilson J. Ornithol. 120: 575–581.

Petry, M. V et al. 2018. Population trends of seabirds at Stinker Point, Elephant Island, Maritime Antarctica. - Antarct. Sci. 30: 220–226.

Ratcliffe, N. et al. 2014. Love thy neighbour or opposites attract? Patterns of spatial segregation and association among crested penguin populations during winter. - J. Biogeogr. 41: 1183–1192.

Raya Rey, A. et al. 2014. Species-specific population trends detected for penguins, gulls and cormorants over 20 years in sub-Antarctic Fuegian Archipelago. - Polar Biol. 37: 1343–1360.

Salton, M. et al. 2019. Population status of the endemic royal penguin (Eudyptes schlegeli) at Macquarie Island. - Polar Biol. 42: 771–781.

Schrimpf, M. B. et al. 2020. Regional breeding bird assessment of the Antarctic Peninsula. - Polar Biol. 43: 111–122.

Strycker, N. et al. 2021. Fifty-year change in penguin abundance on Elephant Island, South Shetland Islands, Antarctica: results of the 2019–20 census. - Polar Biol. 44: 45–56.

Thiebot, J. B. et al. 2013. A Space Oddity: Geographic and Specific Modulation of Migration in Eudyptes Penguins. - PLoS One 8: 1–13.

Trathan, P. N. et al. 2012. Ecological drivers of change at South Georgia: the krill surplus, or climate variability. - Ecography (Cop.). 35: 983–993.

Whitehead, T. O. 2017. Comparative foraging ecology of macaroni and rockhopper penguins at the prince edward islands.

Whitehead, T. O. et al. 2016. Habitat use and diving behaviour of macaroni Eudyptes chrysolophus and eastern rockhopper E. chrysocome filholi penguins during the critical pre-moult period. - Mar. Biol. 163: 1–20.

Woehler, E. J. and Green, K. 1992. Consumption of marine resources by seabirds and seals at Heard Island and the McDonald Islands. - Polar Biol. 12: 659–665.
